# Large-scale achromatic flat lens by light frequency-domain coherence optimization

**DOI:** 10.1038/s41377-022-01024-y

**Published:** 2022-11-11

**Authors:** Xingjian Xiao, Yunwei Zhao, Xin Ye, Chen Chen, Xinmou Lu, Yansen Rong, Junhong Deng, Guixin Li, Shining Zhu, Tao Li

**Affiliations:** 1 National Laboratory of Solid State Microstructures, Key Laboratory of Intelligent Optical Sensing and Manipulations, Jiangsu Key Laboratory of Artificial Functional Materials, College of Engineering and Applied Sciences, Nanjing University, Nanjing, 210093 China; 2 Department of Materials Science and Engineering, Southern University of Science and Technology, Shenzhen, 518055 China

**Keywords:** Micro-optics, Imaging and sensing

## Abstract

Flat lenses, including metalens and diffractive lens, have attracted increasing attention due to their ability to miniaturize the imaging devices. However, realizing a large scale achromatic flat lens with high performance still remains a big challenge. Here, we developed a new framework in designing achromatic multi-level diffractive lenses by light coherence optimization, which enables the implementation of large-scale flat lenses under non-ideal conditions. As results, a series achromatic polymer lenses with diameter from 1 to 10 mm are successfully designed and fabricated. The subsequent optical characterizations substantially validate our theoretical framework and show relatively good performance of the centimeter-scale achromatic multi-level diffractive lenses with a super broad bandwidth in optical wavelengths (400–1100 nm). After comparing with conventional refractive lens, this achromatic lens shows significant advantages in white-light imaging performance, implying a new strategy in developing practical planar optical devices.

## Abstract (web summary)

We developed a new framework in designing centimeter-scale achromatic multi-level diffractive lenses by light frequency-domain coherence optimization, which shows significant advantages in white-light imaging performance over the traditional refractive lens.

## Graphical abstract

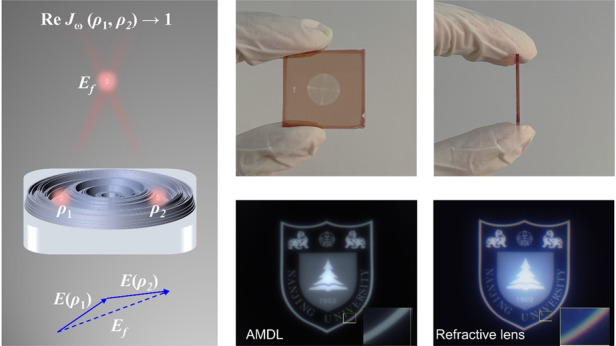

## Introduction

Conventional refractive lens has achieved great success in the area of imaging, metrology, detection, etc^1,2^, but its bulky and heavy characteristics prevent the systems from being compact, lightweight, and miniaturized, which is in high demand for portable and convenient applications^3^. Metalenses, made of subwavelength nano-unit cells with ultrathin, lightweight, and flat architecture, do provide possible solutions that to some extent overcome the shortcomings of conventional diffractive lens with higher focusing efficiency and weak shadow-effect^4–9^. However, metalenses still severely suffer from poor imaging quality under broadband illumination due to serious chromatic aberration^10–13^. Several pioneering works have demonstrated achromatic metalens operating over a broad wavelength range^14–31^. However, according to the fundamental limitations of the achromatic flat lens, the diameter of an ideal achromatic metalens (under the diffraction limit) is restricted by its thickness (the max height each nano-unit can achieve)^18–20,32–34^. As results, the diameters of reported achromatic metalenses with NA ≥ 0.1 in the visible do not exceed 200 μm due to their relatively small thicknesses (<4 μm) up to date. Such miniaturized metalenses are useful in the micro-nano optics or integrated optics (for example, endoscope^7^). However, millimeter- or centimeter-scale metalenses are necessary when it comes to other fields, such as augmented reality, lens modules in cell phones, landscape cameras and telescopes^1,15^. Under theoretical estimation, hundred-micron thickness is needed to access the ideal centimeter scale (5~10 mm) achromatic metalens, which requires a very large aspect ratio (>1000:1) for the constructing nano-units, while the reported largest one is only about 50:1^28,34,35^. Therefore, to access an ideal large-scale achromatic metalens based on the common meta-designs seems to be unachievably difficult.

Fortunately, the multi-level diffractive lens provides an alternative strategy to reach the achromatism by locally controlling the height of each diffractive ring^36–43^. The reported maximum thickness of an achromatic multi-level diffractive lens (AMDL) working in the visible can reach as high as 10 μm, which ensures a relatively large diameter (~1 mm) of the lens^43^. However, such a large thickness is realized with sacrifice in very small numerical aperture (NA = 0.028) corresponding to a large ring width (8 μm), where the aspect ratios of rings can be maintained at a low level. Nevertheless, such a thickness is still far from the one that an ideal centimeter-scale achromatic lens requires. A possible solution would lie in a compromise in designing *a non-ideal broadband achromatic lens* with an achievable thickness and fabrication feasibility. From this point of view, elaborate optimizations in light field coherence at focus and detailed structures are very important and necessary.

In this work, we try to solve the tough problem in two steps. First, we carefully derived the fundamental limits on the comprehensive performance (include size, *NA* and focus efficiency) of a non-ideal AMDL with limited thickness based on the coherence of the light field. Second, we propose an optimization framework to design feasible large-scale AMDLs with performance closed to the upper bound. We substantially validate these results by designing and characterizing five AMDLs of millimeter to centimeter sizes. The optical characterization results prove the effectiveness of our theory and design methods, and demonstrated a relatively high performance of large-scale flat lens in achromatic imaging. The advancement is further confirmed by comparing it with a conventional refractive lens, indicating a major step towards the practical applications.

## Results

### Fundamental limits on performance of non-ideal achromatic flat lens

Previous works have analyzed the fundamental limitations on an ideal achromatic flat lens based on the assumption that the phase profile *φ* provided by lens following the hyperbolic relation^20,32^,

1 $$\varphi (\rho ,\omega ) = - \frac{\omega }{c}(\sqrt {\rho ^2 + F^2} - F)$$

where *ω*, *F*, *ρ* and *c* are the angular frequency, focal length, radial coordinate, and speed of light in vacuum, respectively. The group delay ∂*φ*/∂*ω* can be derived from Eq. (1) and then restriction relations between the size, thickness, working spectrum and numerical aperture (NA) of an ideal achromatic flat lens can be derived^19^. However, when it comes to the *non-ideal achromatic flat lens*, there will exist distortion Δ*φ*(*ρ*, *ω*) in the phase profile as

2 $$\varphi (\rho ,\omega ) = - \frac{\omega }{c}(\sqrt {\rho ^2 + F^2} - F) + \Delta \varphi (\rho ,\omega )$$

**Fig. 1:**
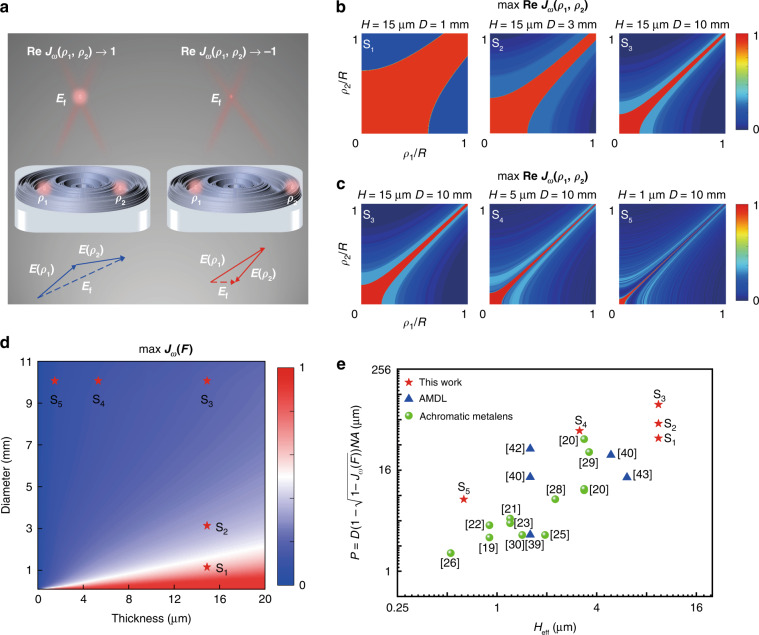
The restriction relation between performance and parameters of AMDLs. **a** Schematics of the coherence of wave distortion in MDL. *E*_*f*_ is the superposition of *E*(*ρ*_1_) and *E*(*ρ*_2_) at focus. The constructive *E*_*f*_ and destructive *E*_*f*_ are denoted as blue dashed lines and red dashed lines, respectively. **b** The distribution of max *J*_*ω*_(*ρ*_1_,*ρ*_2_) with thickness fixed at 15 μm and diameter equal to 1, 3, 10 mm, respectively. **c** The distribution of max *J*_*ω*_(*ρ*_1_,*ρ*_2_) with diameter fixed at 10 mm and thickness equal to 15, 5, 1 μm, respectively. **d** The max *J*_*ω*_(*F*) with respect to different samples in dimensions of diameter and thickness. **e** Comprehensive performance *P* of reported achromatic metalenses and AMDLs with respect to effective thickness *H*_*eff*_

The general form of group delay ∂*φ*/∂*ω* cannot be derived in this case, which means it becomes nearly impossible to work out a quantitative restriction relation based on previous method. To explore the impact of phase distortion, we elaborately deal with this problem from another perspective – the coherence of the light field. The coherence of the light field has been extensively studied in wave optics^44,45^. Achromatic flat lens needs to work within a range of frequencies, so we pay more attention to the coherence in the frequency domain. With analogy to the mutual coherence function in classical optics^45^, the frequency domain mutual coherence function of wave distortion at two coordinates *ρ*_1_ and *ρ*_2_ on the exit pupil (coincide with the lens surface in the single-lens imaging system), denoted as *J*_*ω*_(*ρ*_1_,*ρ*_2_), can be defined as

3 $$J_\omega \left( {\rho _1,\rho _2} \right) = \left\langle {e^{i\Delta \varphi \left( {\rho _1,\omega } \right)}e^{ - i\Delta \varphi \left( {\rho _2,\omega } \right)}} \right\rangle _\omega$$

where 〈〉_ω_ means frequency-averaging operation. The real part of *J*_*ω*_ indicates the degree of the coherence at *ρ*_1_ and *ρ*_2_. If there is no distortion in the whole working spectrum (or equivalently distortion is a constant over the whole radial axis), the real part of *J*_*ω*_ will reach the maximum value +1, which in fact means the sub-waves emitted from *ρ*_1_ and *ρ*_2_ to the focus (denoted as *E*(*ρ*_1_) and *E*(*ρ*_2_)) are totally constructive. If the distortion is large, the real part of *J*_*ω*_ will be closed to the minimum value −1, which means *E*(*ρ*_1_) and *E*(*ρ*_2_) are totally destructive, as is shown in Fig. 1a. Based on propagation of mutual intensity, the normalized coherence function at focus *J*_*ω*_(*F*) can be derived as

4 $$J_\omega (F) = \frac{{F^2}}{{{{\pi}}^2}{R^4}}\mathop {\iint}\limits_\Sigma {J_\omega \left( {\rho _1,\rho _2} \right)} \frac{1}{{r_1r_2}}d\Sigma _1d\Sigma _2$$

where *F* is the focus length, *r*_i_ is the distance from *ρ*_i_ to the focus (i = 1,2), *R* is the radius of the lens and *d*Σ_1_*d*Σ_2_ is a surface element of exit pupil Σ of lens^45^. The achromatic focus performance of the lens relies on the value of *J*_*ω*_(*F*). Generally speaking, *J*_*ω*_(*F*) can be explained as the relative light intensity at focus compared to the background noise on focus plane over the whole working spectrum, and there exists a connection between upper bound of *J*_*ω*_(*F*) and the maximal averaging focus efficiency based on scalar diffraction theory^46^ (see Supplementary S1–4 for more details),

5 $$\max J_\omega (F) \approx \max \frac{{\left\langle {Eff} \right\rangle _\omega }}{{w_{\max }^2}}$$

where *Eff* is focus efficiency defined as the ratio of the optical power within 3 times the full-width at half-maximums (FWHMs) of the point spread function (PSF) on the focal plane over the whole transmitted optical power on the exit pupil^47^, and *w*_max_ is the maximum normalized diameter of PSF in the whole spectrum. In fact, *J*_*ω*_(*ρ*_1_,*ρ*_2_) cannot reach maximum for every two points at exit pupil for a non-ideal lens, and thus maximum focus efficiency will inevitably degrade according to Eqs. (4–5), which indicates performance degradation from an ideal lens to a non-ideal lens is actually due to the reduction in the degree of coherence.

Equation (4) is a general form to describe the relation between the phase distortion Δ*φ*(*ρ*, *ω*) and focus performance for any type of achromatic flat lenses. To quantify the restriction relation, we mainly consider AMDLs in the following, of which the phase profile can be derived based on accumulation of optical path^36^,

6 $$\varphi _{AMDL}(\rho ,\omega ) = \frac{\omega }{c}(n(\omega ) - 1)h(\rho )$$

where *h*(*ρ*) and *n*(*ω*) are radial height distribution (ranging from 0 to maximum thickness *H*) and refractive index, respectively. Firstly, to reveal the restriction of diameter and thickness on the upper bound of coherence at exit pupil (denoted as max Re *J*_*ω*_(*ρ*_1_,*ρ*_2_)) for AMDLs, we perform calculation on 5 different cases based on Eqs. (3) and (6). In each case the AMDL has the same NA (equal to 0.1), working spectrum (400–1100 nm) and is made of the same material (AZ4562, the refractive index is provided in Supplementary S3-1). Figure 1b shows the max Re *J*_*ω*_(*ρ*_1_,*ρ*_2_) of the first three cases (denoted as S_1_-S_3_) with *H* equal to 15 μm and diameters (denoted as *D*) ranging from 1 mm to 10 mm. Figure 1c shows max Re *J*_*ω*_(*ρ*_1_,*ρ*_2_) of last three cases (denoted as S_3_-S_5_) with *D* equal to 10 mm and *H* ranging from 15 to 1 μm. The performance trend is clearly revealed in these results. For a thicker and smaller AMDL (*D* = 1 mm, *H* = 15 μm), high coherence region (red area where max Re *J*_*ω*_(*ρ*_1_,*ρ*_2_) ≈1) covers a large area in (*ρ*_1_, *ρ*_2_) space. As the thickness decreases or diameter increases, the red area shrinks gradually, which indicates the overall coherence of corresponding AMDL will decrease. Secondly, the upper bound of *J*_*ω*_(*F*) with respect to diameter and thickness is calculated based on Eq. (4), with the same fixed NA, working spectrum, and material, as is shown in Fig. 1d. It is clear that max *J*_*ω*_(*F*) has a positive relation to thickness and negative relation to diameter. The max *J*_*ω*_(*F*) in case S_1_-S_5_ is 0.72, 0.43, 0.21, 0.11 and 0.03, respectively, which is marked as five-pointed star in Fig. 1d. More details of the calculation are provided in Supplementary S1-5.

A comprehensive evaluation on AMDL can be derived if the constructive red area is chosen as the only domain of integration in Eq. (4),

7 $$D_{\max } \approx \frac{{4(n_{\max } - 1)H}}{{(1 - \sqrt {1 - \max J_\omega (F)} )NA}}$$

where *n*_max_ is the maximum *n*(*ω*) over the whole spectrum and *D*_max_ is the maximum diameter for given *H*, *NA*, *n*_max_, and max *J*_*ω*_(*F*). Although it is just a rough approximation, Eq. (7) gives us an intuitive insight on the designing trade-offs for AMDL. Increase of the diameter of an AMDL with fixed NA requires an increase in the thickness, otherwise it leads to degradation in the focus performance. It is worth noting that though Eq. (7) is derived for AMDL, it also works for achromatic metalenses approximately, by which the comprehensive performance of an achromatic flat lens can be defined using an index of product $P = D(1 - \sqrt {1 - J_\omega (F)} )NA$. It has taken the size, *NA*, focus performance into account at the same time. The comprehensive performance of some of reported AMDLs and achromatic metalens with respect to the effective thickness *H*_*eff*_ = (*n*_*max*_ *−*1)*H* are summarized in Fig. 1e. Although most achromatic metalenses have shown good performance in achromatic focusing, it is clear that the small lens size (that leads to small field of view) limits the comprehensive performance, which is in fact determined by their low effective thickness. In contrast, by increasing the thickness, the AMDLs show much better comprehensive performances (i.e., an order of magnitude higher than achromatic metalenses). More details of theoretical analysis are provided in Supplementary S1-6 and Table S1.

### Design of AMDLs

Although the analysis of coherence can predict the max *J*_*ω*_(*F*) of an AMDL with specific parameters, it is hard to derive the corresponding optimal height distribution *h*(*ρ*) directly from Eqs. (4–7). Furthermore, considering the feasibility in fabrication, the aspect ratio of *h*(*ρ*) needs to be maintained at a low level in general. The maximum *H* in this work is up to 15 μm, and width of ring Δ is set as 2 μm to satisfy Nyquist−Shannon sampling theorem (Δ ≤ *λ*/2NA). Thus, the aspect ratio may reach 7.5:1, which is very challenge for the grayscale laser lithography technique working in the visible.

**Fig. 2:**
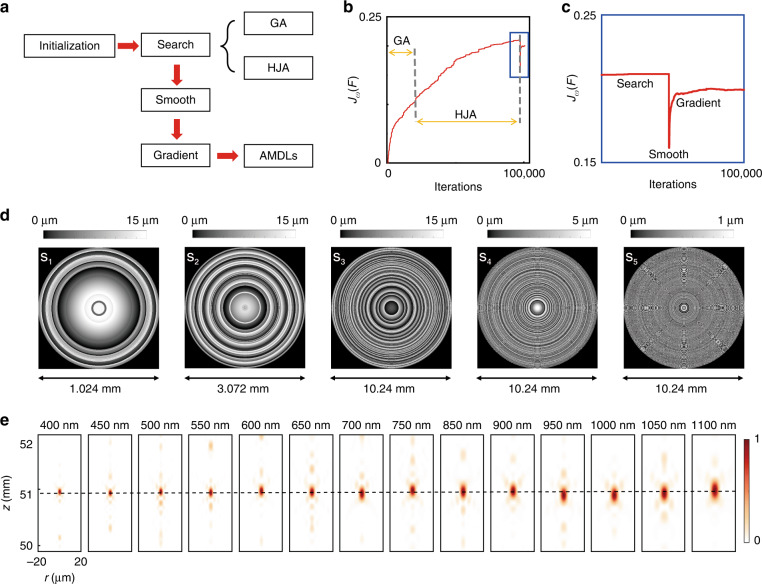
Design and theoretical results of AMDLs. **a** The schematic of the optimization framework. **b** The change of *J*_*ω*_(*F*) over the course of the optimization process. Before the sharp dip is the *Search* operation, where GA and HJA are applied in turn. **c** The details around the sharp dip in **b**. The sharp dip is caused by the *Smooth* operation. **d** Height distribution (2D top view) of samples S_1_-S_5_. **e** Calculated light intensity profiles along propagation axis for sample S_3_ at 14 wavelengths

To overcome these difficulties, an optimization framework is developed as shown in Fig. 2a, which includes three main steps. The first is *Search*, which can yield the optimal height distribution by combining genetic algorithm (GA) and Hook-Jeeves algorithm (HJA)^48–50^ to avoid local minima and speed up the convergence as well. The second is *Smooth*, which can remove the high aspect-ratio structures in the lens. However, *Smooth* will inevitably degrade the performance of the lens. Therefore, we applied the third step – *Gradient* to optimize the samples once more^51^. The design goal is set as maximizing *J*_*ω*_(*F*). Through this optimization framework, we successfully derived the quasi-optimum height distribution of five samples for case S_1_-S_5_, with aspect ratio of most rings less than 2:1. Figure 2b shows *J*_*ω*_(*F*) over the course of the optimization process for sample S_3_. Figure 2c shows the details around the sharp dip in Fig. 2b, which is caused by the *Smooth* operation. Figure 2d shows the height profile of samples S_1_-S_5_ after optimization. We also calculated the diffracted fields along the propagation axis z for these samples based on Rayleigh-Sommerfeld diffraction theory to confirm the achromatic properties^46^. Figure 2e shows the diffracted fields of S_3_ at 14 wavelengths between 400 and 1100 nm. It is clear that light focuses at the same point (*z* = 50.9 mm) for all the wavelengths. More details about optimization process and theoretical results of other samples are provided in Supplementary S2.

### Fabrication and characterizations of AMDLs

**Fig. 3:**
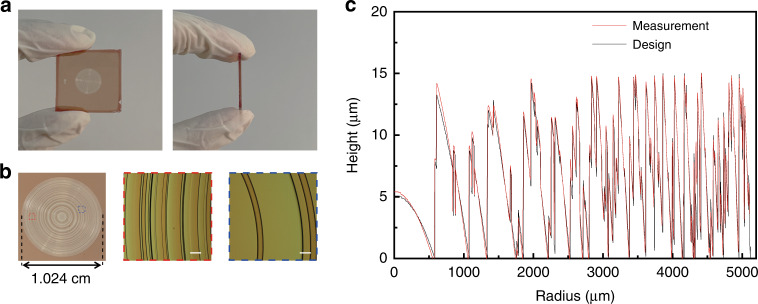
Fabricated AMDL and characterization. **a** Photographs of fabricated samples S_3_, the left one and right one are top and side views, respectively. **b** Local view of the whole AMDL (left) and zoom-in optical image (mid and right). Scale bar, 100 μm. **c** The designed (black line) and measured (red line) height distribution, respectively

AMDL samples S_1_-S_5_ are fabricated by using the grayscale laser lithography technique (see more details in “Methods”). The top view and side view of a typical AMDL are shown in Fig. 3a. Figure 3b particularly shows the photograph of sample S_3_ and its zoom-in details, whose height profile is characterized by the step profiler with comparison to the theoretical design, as shown in Fig. 3c. The average fabrication error is <150 nm over the whole lens (<1%), showing extremely good agreement. Details of other samples are provided in Supplementary S3-2.

**Fig. 4:**
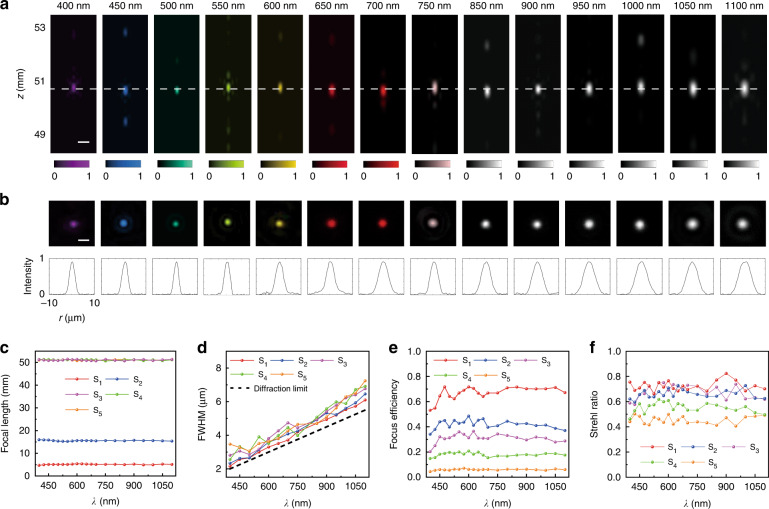
Measured achromatic focusing characteristic of AMDLs. **a** Experimental light intensity profiles of sample S_3_ at 14 different incident wavelengths. Scale bars, 15 μm. **b** Normalized intensity profiles along the white dashed lines of **a**. Scale bars, 10 μm. **c** Experimentally measured focal length shifts of samples S_1_-S_5_. **d** Experimentally measured FWHM of PSF of samples S_1_-S_5_ and the diffraction limited FWHM (black dashed dots). **e** Measured focus efficiencies of samples S_1_-S_5_. **f** Experimentally measured Strehl ratios of samples S_1_-S_5_

The focusing performance of AMDLs was experimentally examined by a home-built optical setup, where a super-continuum laser (Fianium, Super-continuum 4 W, the spectrum is provided in Supplementary S3-5) with a beam expander was used to generate large area uniform beam (with diameter > 1 cm). Figure 4a shows the measured cross-section of intensity profiles at 14 different wavelengths for sample S_3_ with a designed focal length *f* = 50.9 mm, from which it is observed that the brightest spots are almost located at the designed position (white dash line) for all wavelengths. The measured 2D (top) and 1D (bottom) point spread function (PSF) at each wavelength at the focal plane (dashed line in Fig. 4a) are displayed in Fig. 4b with the intensity normalized to 0~1, showing good coincidence with theoretical Gaussian distribution and confirming the broadband achromatism very well. Figure 4c shows the experimentally measured focal lengths of samples S_1_-S_5_, where the maximum discrepancy is found less than 2%. The experimentally measured FWHM of the PSF at each wavelength are plotted in the Fig. 4d, which is slightly larger than the diffraction limit (*λ*/2*NA*, black dashed lines). Figure 4e shows the measured focus efficiency of S_1_-S_5_ at each wavelength. The definition of focus efficiency is the same as the one used in analyzation above. The average efficiency is about 68%, 42%, 31%, 18%, 5% for samples S_1_-S_5_, respectively. Based on Eq. (5), we can estimate the coherence *J*_*ω*_(*F*) of samples S_1_-S_5_, which is 0.61, 0.34, 0.18, 0.085, 0.014, respectively, in good coincidence with our theoretical prediction (Fig. 1d). The focus performance can be further quantified by measuring Strehl ratios, as are shown in Fig. 4f. The Strehl ratio is calculated based on the measured 2D PSF at each wavelength^43^. The average Strehl ratio of sample S_1_ is 0.7252, which is close to the diffraction limit (~0.8), while that of S_5_ is only 0.4545. More details about focus performance of other samples are provided in Supplementary S3-6.

**Fig. 5:**
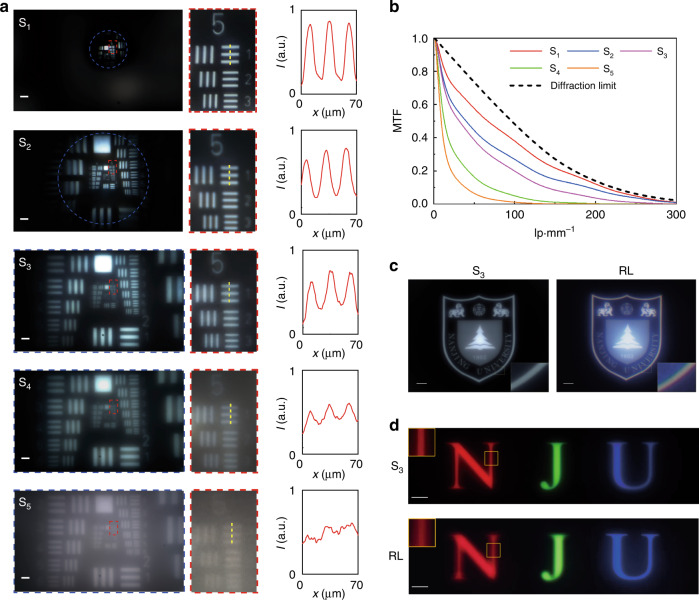
Imaging performance of AMDLs. **a** Image results of 1951 USAF resolution test chart taken from samples S_1_ ~ S_5_. The whole images captured by CCD (left column). Scale bar, 250 μm; zoom-in images of group 5 element 1~3 (middle column); the light intensity (denoted as I) along the yellow dashed lines (right column). **b** The broadband MTF of samples S_1_-S_5_ and diffraction limited MTF (black dashed line). **c** Image of Nanjing University logo taken from sample S_3_ and a refractive lens. Zoom-in image shows the edge details of the logo. Scale bars, 2 mm. **d** Images of three colorful letters (N, J, U) taken from sample S_3_ and a refractive lens. Zoom-in image shows the edge details of the letter R. Scale bars, 4 mm

Afterwards, the comprehensive imaging performance of these AMDLs are systematically analyzed. First, the 1951 United State Air Force (USAF) resolution test chart was used as imaging target, illuminated by a broadband illumination source (HL100S, color temperature = 3200 K), and a charge coupled device (CCD, Blackfly S BFS-U3-200S6C) was employed to capture the image. The left column in Fig. 5a shows the whole images taken from S_1_-S_5_ captured by the CCD under white light illumination. In each figure, the closed blue dashed lines denote the field of view (FOV) and the red dashed lines denote the images of group 5 element 1~3. It is clear that due to the achromatic design, there exists no color blur effect in all the images taken by AMDLs, which is obvious in the cases of chromatic refractive lens and Fresnel lens (shown in Supplementary S3-7). The middle column in Fig. 5a displays the details of group 5 element 1~3, whose intensity along the yellow dashed lines is shown in the right column in Fig. 5a. Using *I*_max_ and *I*_min_ to denote the maximum and minimum intensity along the yellow dashed lines, the image contrast, defined as (*I*_max_ – *I*_min_)/(*I*_max_ + *I*_min_), is 0.66, 0.44, 0.34, 0.15, 0.06 for images taken by S_1_ to S_5_, respectively. It is clear that the image contrast has a positive relation with the coherence *J*_*ω*_(*F*). These results indicate that *J*_*ω*_(*F*) cannot be too low (<0.1), otherwise the corresponding image contrast will be extremely low and even unable to resolve the image details (e.g., sample S_5_). We further characterized the broadband Modulation Transfer Function (MTF) under white light illuminance of each sample, as the results shown in Fig. 5b. It shows very high value of S_1_ (red dashed line) that is close to the diffraction-limited one (black dashed line) while the MTF of S_5_ (orange dashed line) is far below it. Although the results show that S_1_ owns the best focus performance, it is restricted by the relatively small FOV preventing it from widely practical applications. In fact, considering the comprehensive performance, sample S_3_ would be the best one among samples S_1_-S_5_ which has already been indicated in Fig. 1e.

More advantages in imaging performance of the AMDL are further evaluated by comparing with a conventional refractive lens (LE1234, denoted as RL). Figure 5c shows the images of Nanjing University logo (16 mm × 20 mm, generated by a projector) taken from sample S_3_ and RL. Although the image taken from sample S_3_ is a little bit darker due to relatively lower efficiency, there is no color blurred effect, which is very obvious in the case for RL (see the corresponding zoom-in edge images of both). Figure 5d shows the imaging results of R, G, B letters (generated by a projector), where all three letters can be clearly imaged on the same plane for AMDL. However, the image of R and G will be blurred when B is clearly imaged for RL due to the chromatic aberration (see the corresponding zoom-in edge images at the upper-left corner). Considering its thin and lightweight, such an AMDL is very promising for portable and convenient applications in the nearly future.

## Discussion

By now, we are aware of the importance of the thickness in AMDL design, which is also in coincidence with the thicker achromatic metalens design for improved performance^28^. Although AMDL can reach larger thickness than metalens, there is still a great distance to achieve the level required by an ideal macro-size lens, due to the extremely difficulty in manufacturing. Our experimental results highlight the significance of our theory and method that provide a guidance to derive the optimal thickness for an AMDL with given diameter and NA. In addition, it should be mentioned that the working bandwidth Δ*λ* is also an important parameter in the design of AMDLs. However, the restriction relation between Δ*λ* and other parameters is not clear in analytical form Eq. (7) due to the approximations applied in the derivation of this equation (see Supplementary Material S1-6). In fact, if the thickness *H*, diameter *D* and *NA* of an AMDL are fixed, the focus performance will be improved as working bandwidth Δ*λ* decreases through numerical simulation based on Eq. (4), which is shown in Supplementary S2-6. A centimeter-scale AMDL with larger NA (≥0.3), narrower working bandwidth (450 nm–680 nm) and relatively good performance is also revealed theoretically and provided in Supplementary S2-6. To be noted, further improvement on comprehensive performance can be made by incorporating AMDL with matured imaging process algorithms. As a preliminary attempt, we reconstruct high-contrast images from the raw images taken by AMDLs based on a simple built-in image processing algorithm in our cell phone, and find the maximum improvement in images taken by S_3_, which is provided in Supplementary S3-9. These results also highlight the necessity of achromatic design and reservation of details in raw images. It is very hopeful that the performance of the AMDL can be further enhanced to meet the real application by incorporating with advanced imaging processing, like the deep learning^52^, etc.

In conclusion, we elaborately derived the fundamental limitations existed in *non-ideal AMDLs* by analyzing the coherence of the light field, and developed an optimization framework to realize a large-scale AMDL with relatively high performance, large thickness and small aspect ratio of ring structures. As a proof of concept, a series of AMDLs were designed and demonstrated in super-broad wavelength range (400~1100 nm), among which the maximum diameter and thickness of AMDLs reach 1 cm and 15 μm, respectively. Considering the size, efficiency and numerical aperture, such an AMDL presented an extremely high comprehensive performance, while no existing achromatic flat lens is reported approaching a comparable value like this (Fig. 1e). Our strategy is helpful to evaluate the upper bound of an achromatic flat lens to applicable level with the progress of manufacturing, which is very promising to be applied to the conventional imaging systems.

## Materials and methods

### Refractive index measurement

A thin positive photoresist (AZ4562) layer with thickness of ~400 nm was spin-coated onto a silicon substrate. Then, the wavelength dependent (400–1100 nm) refractive index was measured by using the spectroscopic ellipsometer.

### Sample fabrication

A thin positive photoresist (AZ4562) layer with a thickness of ~25 µm was spin-coated onto a 1.1 mm thick glass substrate. The photoresist was baked at 100 °C for 5 min. The grayscale laser lithography technique was used to transfer the grayscale information of the AMDL to the spatially dependent exposure on the photoresist layer. The wavelength of the laser used for grayscale lithography is 405 nm. After developing the exposed photoresist layer, the AMDL with pre-defined three-dimensional morphology were realized on glass substrate.

## Supplementary information

Large-scale achromatic flat lens by light frequency-domain coherence optimization
