# Supplementary material for: Large-scale achromatic flat lens by light frequency-domain coherence optimization
Source: Light Sci Appl. 2022 Nov 11;11:323. doi: 10.1038/s41377-022-01024-y (PMC9649754; doi:10.1038/s41377-022-01024-y)
Supplement: Supplementary file 1 — Large-scale achromatic flat lens by light frequency-domain coherence optimization [file 41377_2022_1024_MOESM1_ESM.docx]

Supplementary Materials for

**Large-scale achromatic flat lens by light frequency-domain coherence optimization**

Xingjian Xiao^1^, Yunwei Zhao^1^, Xin Ye^1^, Chen Chen^1^, Xinmou Lu^2^, Yansen Rong^2^, Junhong Deng^2^, Guixin Li^2^*, Shining Zhu^1^, Tao Li^1^*

*^1^National Laboratory of Solid State Microstructures, Key Laboratory of Intelligent Optical Sensing and Manipulations, Jiangsu Key Laboratory of Artificial Functional Materials, College of Engineering and Applied Sciences, Nanjing University, Nanjing, 210093, China.*

*^2^Department of Materials Science and Engineering, Southern University of Science and Technology, Shenzhen, 518055, China.*

**Corresponding authors: ligx@sustech.edu.cn,* [*taoli@nju.edu.cn*](mailto:taoli@nju.edu.cn)

### Details of theoretical analyzation on non-ideal achromatic flat lens

### S1-1. The propagation of coherence of the light field in frequency domain

The conventional mutual coherence evaluated for *τ* = 0 between field *E* at *ρ*_1_ and *E* at *ρ*_2_ is defined as^1^

()

where * denotes complex conjugation and _t_ means time-averaging operation. The mutual intensity at focus, denoted as *J*(*F*), can be calculated based on the propagation of mutual intensity^1^,

(2)

where Σ denotes the exit pupil of the AMDL, (i=1,2). Considering time-independent field *E* and assuming that lenses provide phase-only modulation,

()

The definition of Δ*φ*(*ρ*, *ω*) is provided in the main text. *J*(*F*) will reach maximum π^2^*R*^4^/*λ*^2^*F*^2^ when Δ*φ*(*ρ*_1_, *ω*) = Δ*φ*(*ρ*_2_, *ω*) for every (*ρ*_1_,*ρ*_2_) in Σ. (under paraxial approximation *R*<<*F*). Then the normalized *J*(*F*) is

()

Averaging both sides of Eq. S5 with respect to frequency and the propagation of coherence of the light field in Frequency domain can be yielded

()

### S1-2. Detailed analyzation of *J*_ω_(*ρ*_1_, *ρ*_2_)

Based on Eq. (2), Eq. (3) and Eq. (6) in the main text, real part of *J*(*ρ*_1_, *ρ*_2_) can be calculated as follows

$\mathrm{Re}J_{\omega}(\rho_{1},\rho_{2})=\frac{1}{\omega_{2}-\omega_{1}}\int_{\omega_{1}}^{\omega_{2}} \cos(\frac{\omega}{c}[(n-1)(h(\rho_{1})-h(\rho_{2}))+(r_{1}-r_{2})])d\omega$ ()

where *ω*_1_, *ω*_2_ are the lower and upper bound of the working spectrum. Due to the fact that cos(x) ∈ [-1,1] for every x∈ℝ, Re *J*(*ρ*_1_, *ρ*_2_) ranges from -1 to 1 and achieves the maximum 1 when

$\cos(\frac{\omega}{c}[(n-1)(h(\rho_{1})-h(\rho_{2}))+(r_{1}-r_{2})])\equiv1\Rightarrow\left\{\begin{matrix} \frac{\omega}{c}(n-1)h(\rho_{1})=-\frac{\omega}{c}r_{1}+C(\omega) \\ \frac{\omega}{c}(n-1)h(\rho_{2})=-\frac{\omega}{c}r_{2}+C(\omega) \end{matrix}\right.$ ()

where *C*(*ω*) is a variable only depending on *ω*. Eq. S8 means the phase at *ρ*_1_ and *ρ*_2_ simultaneously obeys the hyperbolic distribution in the whole achromatic bandwidth, or the sub-waves emitted from at *ρ*_1_ and *ρ*_2_ to the focus are constructive. Re *J*_ω_(*ρ*_1_, *ρ*_2_) achieves the minimum -1 when

$\cos(\frac{\omega}{c}[(n-1)(h(\rho_{1})-h(\rho_{2}))+(r_{1}-r_{2})])\equiv-1\Rightarrow\left\{\begin{matrix} \frac{\omega}{c}(n-1)h(\rho_{1})=-\frac{\omega}{c}r_{1}+C(\omega) \\ \frac{\omega}{c}(n-1)h(\rho_{2})=-\frac{\omega}{c}r_{2}+C(\omega)-\pi\end{matrix}\right.$ ()

which means the phase at *ρ*_1_ obeys the hyperbolic distribution while the phase at *ρ*_2_ obeys the same hyperbolic distribution minus π, or the sub-waves emitted from at *ρ*_1_ and *ρ*_2_ to the focus are destructive.

### S1-3. The physical explanation about impacts of *H* and *D* on *J*_ω_(*F*)

Increasing the thickness H provides more dispersion range for phase compensation, and thus gives rise to more choices in the rings profiles (*h*(*ρ*) distributions) to reach a higher max *J_ω_*(*F*). However, if the diameter *D* increases, a larger phase compensation is needed, which will inevitably lead to more incomplete constructive interference for all diffractive rings if the H is fixed. Therefore, the max *J_ω_*(*F*) will decrease.

### S1-4. The relation between the upper bound of coherence at focus and maximal averaging focus efficiency

We assume that light distribution on focus plane approximatively obeys Airy disc, which can be written as^2^:

$I(\rho,\omega)=I_{0}(\omega)\left(\frac{2J_{1}(\pi\rho/w)}{\pi\rho/w}\right)^{2}$ ()

where *I*_0_ is the peak value of PSF, *w* is the diameter of focus and *J*_1_ is the first order Bessel function. Then the optical power within PSF (usually defined as a circle area with diameter equal to 3*w*) on the focal plane is

$\int_{0}^{2\pi} \int_{0}^{3w} I(\rho,\omega)\rho d\rho d\theta\propto I_{0}(\omega)w^{2}(\omega)$ ()

Thus, focus efficiency can be estimated as *I*_0_*w*^2^ where *w* is normalized by diameter of diffraction limited focus disc (*λ*/2*NA*, full width of half maximum, strictly speaking). The average value of *Eff* over the achromatic spectrum is

$\text{ }\left\langle Eff\right\rangle_{\omega}=\left\langle I_{0}w^{2}\right\rangle_{\omega}=\left\langle\frac{w^{2}(\omega)}{I_{std}(\omega)}\left|\int_{0}^{R} E(\rho,\omega)G(\rho,\omega)\rho d\rho\right|^{2}\right\rangle_{\omega}$ ()

where *G* is the free-space Green’s function along radial axis and *I*_std_(ω) is the peak value of focus for an ideal lens. If *G* is chosen as the second Rayleigh-Sommerfeld solution^2^, the maximum averaging focus efficiency is

$\text{ max}\left\langle Eff\right\rangle_{\omega}\approx\frac{F^{2}w_{^{\max}}^{2}}{\pi^{2}R^{4}}\max\int_{0}^{R} \int_{0}^{R} J_{\omega}(\rho_{1},\rho_{2})\frac{\rho_{1}\rho_{2}}{r_{1}^{2}r_{2}^{2}}d\rho_{1}d\rho_{2}\approx\max J_{\omega}(F)w_{^{\max}}^{2}$ ()

Then the upper bound of *J*(*F*) can be estimated as

$\text{max }J_{\omega}(F)\approx\text{max}\frac{\left\langle Eff\right\rangle_{\omega}}{w_{\max}^{2}}$ ()

It should be mentioned that though max *J_ω_*(*F*) seems to be proportional to the focus efficiency in Eq. S13, the relation between max *J_ω_*(*F*) and <*Eff*> is actually not linear. In fact, *w* is not a constant as max *J_ω_*(*F*) changes. As is shown in the experimental results (see Fig.4d in the main text), *w* will increase slightly as max *J_ω_*(*F*) decreases, which is probably due to the decrease of diffracted light with high spatial frequency at focus.

### S1-5. Calculation of max *J_ω_*(*ρ*_1_, *ρ*_2_) and max *J_ω_*(*F*)

Based on Eq. (3) and Eq. (6) in the main text, *J_ω_*(*ρ*_1_,*ρ*_2_) is a functional of *h*(*ρ*_1_) and *h*(*ρ*_2_) for AMDL. Thus, the upper bound of coherence at exit pupil max *J_ω_*(*ρ*_1_,*ρ*_2_) can be expressed as

$\max J_{\omega}(\rho_{1},\rho_{2})=\max_{\begin{matrix} h(\rho_{i})\in[0,H] \\ \text{ }i=1,2 \end{matrix}} \mathrm{Re}J_{\omega}(\rho_{1},\rho_{2})$ ()

max *J_ω_*(*ρ*_1_,*ρ*_2_) at every (*ρ*_1_, *ρ*_2_) can be calculated alone by traversal algorithm in a relatively short time, which means for every (*ρ*_1_, *ρ*_2_) we take all the possible value (after discretization) of *h*(*ρ*_1_), *h*(*ρ*_2_) into Eq. S14 and then choose the maximum one. Similarly, max *J_ω_*(*F*) can be expressed as

$\max J_{\omega}(F)\approx\frac{F^{2}}{\pi^{2}R^{4}}\iint_{\Sigma} \left[\max_{\begin{matrix} h(\rho_{i})\in[0,H] \\ \text{ }i=1,2 \end{matrix}} \mathrm{Re}J_{\omega}\left(\rho_{1},\rho_{2}\right)\right]\frac{1}{r_{1}r_{2}}d\Sigma_{1}d\Sigma_{2}$ ()

Due to the fact that *J*(*ρ*_1_, *ρ*_2_) is not always independent at two different arguments (*ρ*_1_, *ρ*_2_) and (*ρ'*_1_, *ρ'*_2_), we exchange the order of max operator and integration in Eq. S15 for decoupling. The right side of Eq. S15 is in fact an upper bound of max *J_ω_*(*F*) and thus also represents the upper bound (not supremum) of performance an AMDL with specific parameters.

### S1-6. Derivation of analytic restriction relations

To yield the analytic form of restriction relation, we should make several approximations. First, we only consider the region where max *J_ω_*(*ρ*_1_,*ρ*_2_) =1 in the integration in Eq. S15. Second, we apply paraxial approximation (*R*<<*f*). Third, we ignore the dispersion of refractive index *n* (the dispersion is actually very small). The first approximation comes from the fact that the constructive area contributes most to the integral in Eq. S15, as is represented in Fig. 1b~1c. Based on Eq. S6, the region where max *J_ω_*(*ρ*_1_,*ρ*_2_) =1 is

$\left.\begin{matrix} \frac{\omega}{c}[(n-1)(h(\rho_{1})-h(\rho_{2}))+(r_{1}-r_{2})]=0 \\ \forall\text{ }0\leq h(\rho_{i})\leq H,\text{ }i=1,2 \end{matrix}\right\}\Rightarrow\left|r_{1}-r_{2}\right|\leq(n-1)H$. ()

Then max *J_ω_*(*F*) can be yielded

$\max J_{\omega}(F)\approx\left\{\begin{matrix} \frac{4(n-1)Hf}{R^{2}}-\frac{4(n-1)^{2}H^{2}f^{2}}{R^{4}} & \begin{matrix} R\geq\sqrt{2(n-1)Hf} \\ \end{matrix} \\ 1 & R<\sqrt{2(n-1)Hf} \end{matrix}\right.$ (17)

When $R<\sqrt{2(n-1)Hf}$, *J_ω_*(*F*) reaches maximum 1, which in fact is equivalent to phase profile following hyperbolic distribution over the whole working spectrum. In that case,

$D_{\max}\approx\frac{4(n_{\max}-1)H}{NA}$ (18)

where *D*_max_ is the maximum diameter of the AMDL for given *H*, *NA*, *n*_max_ and max *J_ω_*(*F*). When $R\geq\sqrt{2(n-1)Hf}$,

$D_{\max}\approx\frac{4(n_{\max}-1)H}{(1-\sqrt{1-\max J_{\omega}(F)})NA}$ ()

It is worth noting that Eq. S19 is just an approximate result and will be accurate only when *J_ω_*(*F*) is closed to 1. In addition, it should be mentioned that the first and third approximations have removed the effect of working bandwidth Δ*λ* to max *J*_ω_(*F*). However, there do exist restriction relations between working bandwidth Δ*λ* and max *J*_ω_(*F*), which can only be yielded through numerical calculation and is shown in S2-6. Furthermore, if the meta-unit is treated as a waveguide, the phase profile provided by achromatic metalens can be written as^3^

()

Substitute Eq. S20 into Eq. S15 and a restriction relation similar to Eq. S19 can also be derived.

**Table S1**. Parameters of reported broadband achromatic metalenses and AMDLs.

| Ref | D  (μm) | H  (μm) | λ_min_  (μm) | λ_max_  (μm) | NA | Refractive index_n | H*(n-1) | J_ω_(F) | P |
| --- | --- | --- | --- | --- | --- | --- | --- | --- | --- |
| [19] | 25.72 | 0.6 | 0.47 | 0.67 | 0.2 | 2.5 | 0.9 | 0.7~0.74 | 2.5 |
| [20] | 100 | 1.4 | 1.2 | 1.4 | 0.88 | 3.4 | 3.36 | 0.57~0.67 | 37.4 |
| [20] | 100 | 1.4 | 1.2 | 1.65 | 0.24 | 3.4 | 3.36 | 0.56~0.62 | 9.2 |
| [20] | 200 | 1.4 | 1.2 | 1.65 | 0.13 | 3.4 | 3.36 | 0.55~0.6 | 9.6 |
| [21] | 50 | 0.8 | 0.4 | 0.66 | 0.106 | 2.5 | 1.2 | 0.93~0.96 | 4.2 |
| [22] | 26.4 | 0.6 | 0.46 | 0.7 | 0.2 | 2.5 | 0.9 | 0.86~0.89 | 3.5 |
| [23] | 21.6 | 0.8 | 0.4 | 0.66 | 0.216 | 2.5 | 1.2 | 0.92~0.96 | 3.7 |
| [25] | 20 | 3.9 | 1 | 1.8 | 0.27 | 1.5 | 1.95 | 0.71~0.75 | 2.7 |
| [26] | 20 | 0.35 | 0.64 | 1.2 | 0.12 | 2.5 | 0.53 | 0.86~0.9 | 1.6 |
| [28] | 30 | 1.5 | 0.65 | 1 | 0.24 | 2.5 | 2.25 | 0.95~1 | 7.2 |
| [29] | 490 | 1.5 | 1.3 | 1.7 | 0.238 | 3.4 | 3.6 | 0.34~0.4 | 26.3 |
| [30] | 20 | 2.5 | 0.45 | 1.7 | 0.27 | 1.57 | 1.42 | 0.62~0.75 | 2.7 |
| [30] | 200 | 2.5 | 0.45 | 1.7 | 0.04 | 1.57 | 1.42 | 0.44~0.53 | 2.5 |
| [39] | 370 | 2.6 | 0.45 | 0.75 | 0.18 | 1.61 | 1.59 | 0.06~0.08 | 2.7 |
| [40] | 2500 | 2.6 | 0.44 | 0.66 | 0.05 | 1.61 | 1.59 | 0.17~0.2 | 13.2 |
| [40] | 2500 | 8 | 0.44 | 0.66 | 0.05 | 1.61 | 4.88 | 0.27~0.35 | 24.2 |
| [42] | 3145 | 2.6 | 0.45 | 1 | 0.3 | 1.61 | 1.59 | 0.03~0.06 | 28.7 |
| [43] | 992 | 10 | 0.45 | 15 | 0.0275 | 1.61 | 6.1 | 0.67~0.73 | 13.1 |
| S_1_ | 1024 | 15 | 0.4 | 1.1 | 0.1 | 1.63 | 9.45 | 0.61 | 38.2 |
| S_2_ | 3072 | 15 | 0.4 | 1.1 | 0.1 | 1.63 | 9.45 | 0.34 | 58.2 |
| S_3_ | 10240 | 15 | 0.4 | 1.1 | 0.1 | 1.63 | 9.45 | 0.18 | 97.0 |
| S_4_ | 10240 | 5 | 0.4 | 1.1 | 0.1 | 1.63 | 3.15 | 0.09 | 44.0 |
| S_5_ | 10240 | 1 | 0.4 | 1.1 | 0.1 | 1.63 | 0.63 | 0.014 | 7.2 |

**Table S1** shows the parameters of some AMDLs and broadband achromatic metalenses. The first column is the reference number in the main text. The estimation of *J_ω_*(*F*) is based on Eq.(5) (*J_ω_*(*F*) = *Eff*_ω_/*w_max_*^2^). It should be mentioned that the measured efficiency in most works is the total efficiency, which considers both focus efficiency and other factors, such as Fresnel reflection on the interface, polarization conversion and etc. . For ease of analyzation, we only consider the effect of Fresnel reflection under normal incidence and polarization conversion (assuming it equals to 0.5), and derive the focus efficiency as follows

$\begin{matrix} Eff\text{ }=\text{ }Measured\text{ }total\text{ }efficiency\times\frac{1}{PB}\times\frac{1}{T} \\ T=1-\left(\frac{n-1}{n+1}\right)^{2}\text{ }PB=\left\{\begin{matrix} 1 & polarization\text{ }insensitive \\ 0.5 & polarization\text{ }sensitive \end{matrix}\right. \end{matrix}$ ()

On the other hand, the w_max_ is extracted from the figure in most works. To be more accurate, we estimate a range of w_max_ in each work and thus the corresponding *J_ω_*(*F*) becomes a range. The comprehensive performance P is calculated by the largest *J_ω_*(*F*).

### Details of design method and theoretical results of AMDLs

### S2-1. Formalization of the optimization problem in designing AMDLs

According to the main text, the optimization problem can be written as

$\begin{matrix} \max_{h} J_{\omega}(F) \\ st:0\leq h(\rho_{i})\leq H \\ i=1,2...,N \end{matrix}$ ()

where *H* is the maximum of the height, *ρ_i_* is the radial axis of the *i*^th^ rings and *N* is the total number of the rings. In our design, the possible values of *h* are discretized into 32, 64, and 192 gray-levels for H = 1μm, 5 μm, 15 μm, with every level Δ*h* equal to 31 nm, 78 nm, 78 nm, respectively. Considering that the refractive index *n* equals to 1.63 on average, the accumulated optical path of every level equals to 19.5 nm, 49 nm, 49 nm, which is no more than 0.125 waves even for the smallest wavelength (400 nm), so it can provide effective modulation for every wavelength from 400 nm to 1100 nm. *N* is set as 256, 768, 2560 for D = 1.024mm, 3.072mm, 10.24mm, with width of ring equal to 2 μm, respectively. Then the height profile can be written as *h*(*ρ_i_*) = ***m***(*i*)Δ*h,* where ***m***(*i*) is the gray-level at *ρ_i_* and ranges from 1 to *M*. Now the final form of optimization problem can be written as

$\begin{matrix} \max_{m} J_{\omega}(F) \\ st:0\leq m(i)\leq M \\ i=1,2...,N \end{matrix}$ ()

In initialization, the initial ***m*** is set as a random vector with each element ranges from 0 to *M*.

### S2-2. Details of optimization algorithms used in the framework

There are three optimization algorithms used in the framework, including GA, HJA and Gradient Decent. GA and HJA are used in step *Search*, and Gradient Decent is used in step *Gradient*.

A conventional binary GA is used in the first main step^4^. Before optimization, the distribution *m* needs to be encoded in binary format. Every component of *m* can be encoded into 000000~100000, 0000000~1000000, 00000000~11000000 for maximum gray-levels equal to 32, 64, 192, respectively. Therefore, the *m* can be expressed as a binary vector with length equal to 6*N*, 7*N*, 8*N*. Each binary vector is treated as an individual in the population in GA. In optimization, if some individuals exceed the maximum gray-levels in the cross or mutation operations, these individuals will be set as the maximum gray-levels instead.

The flow chart of Hook-Jeeves Algorithm (HJA)^5^ is shown in Fig.S1, where ***m***^(i)^ is the gray-level distribution ***m*** after *i*^th^ epochs, α is the accelerated factor, *d* is the step size, *f* is the objective function (*J_ω_*(*F*) in this work), ***Y*** is an intermediate variable and ***e***_i_ is the *i*^th^ nature base vector (*i*^th^ vector component equals to 1 while other components equal to 0). Generally speaking, in the *j*^th^ epoch, the *j*^th^ element of ***Y***^(j)^ will increase and decrease *d* to get two new FOMs, respectively. If one of these new FOMs is better than the old one, the corresponding change in ***Y***^(j)^ will go into effect. When stepping into the local optima, the step *d* = [*d*/2], where [] denotes least integer function. There is also an acceleration process, which is denoted as ***Y***^(1)^ = ***m***^(k+1)^ + [*α*(***m***^(k+1)^ - ***m***^(k)^)]. When d≤1, the optimization will terminate and return the final distribution ***m****.

The flow chart of Gradient Descent (GD)^6^ is shown in Fig. S2, where ***m***^(i)^ is the distribution ***m*** after *i*^th^ epochs, *α* is the step length, *δ* is the cut-off step length and *f* is the objective function. When reach cut-off condition, the integer portion of ***m***^(i)^ is taken as the final distribution ***m****.

The flow chart of the combination of GA and HJA (denoted as GA+HJA) is shown in Fig. S3. Due to the fact that it’s hard to know when to stop GA and apply HJA, the optimization process is divided into *s* blocks. In each block (denoted by black dashed square), the epoch of GA is set as *p*, and the initialized distribution of GA is the result ***m**** of GA in last block, while the initialized distribution of HJA is the result of GA in this block. The final distribution (denoted as ***m**^(1)^**, ***m**^(2)^**, … ***m**^(s)^**) is the result of HJA in each block. The whole epoch of GA+HJA (*s∙p*) is much smaller than that of GA alone, and the process of HJA and GA can iterate parallelly. Thus, the whole computation time of GA+HJA is shorter than that of GA alone.


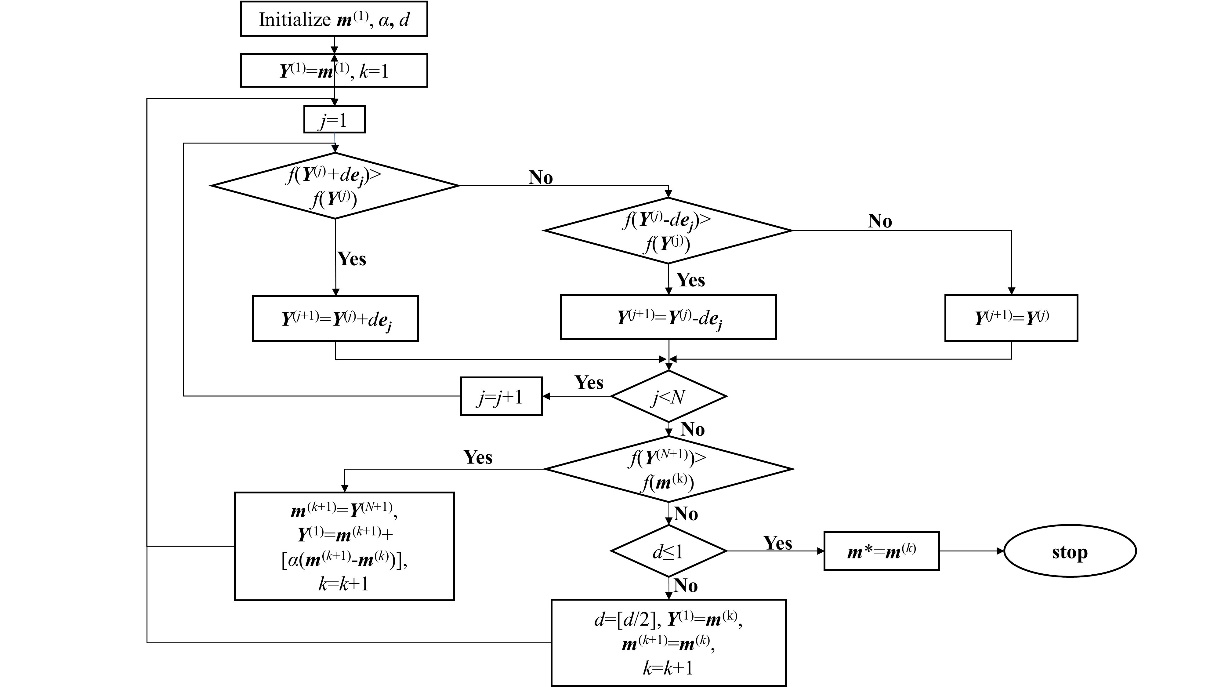


**Fig. S1. The flow chart of** **Hook-Jeeves algorithm.**

**
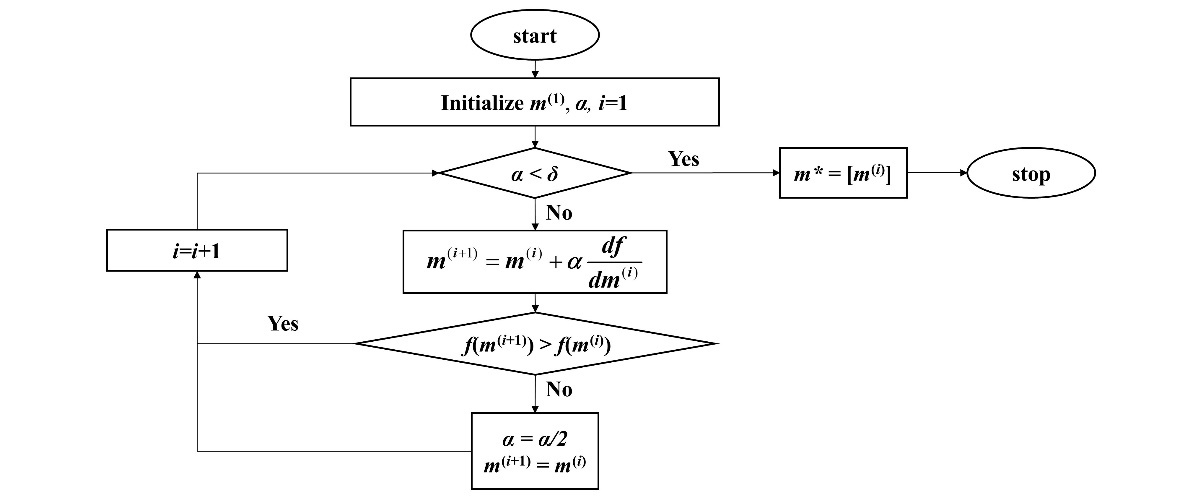
**

Fig. S2. The flow chart of Gradient Descent.

**
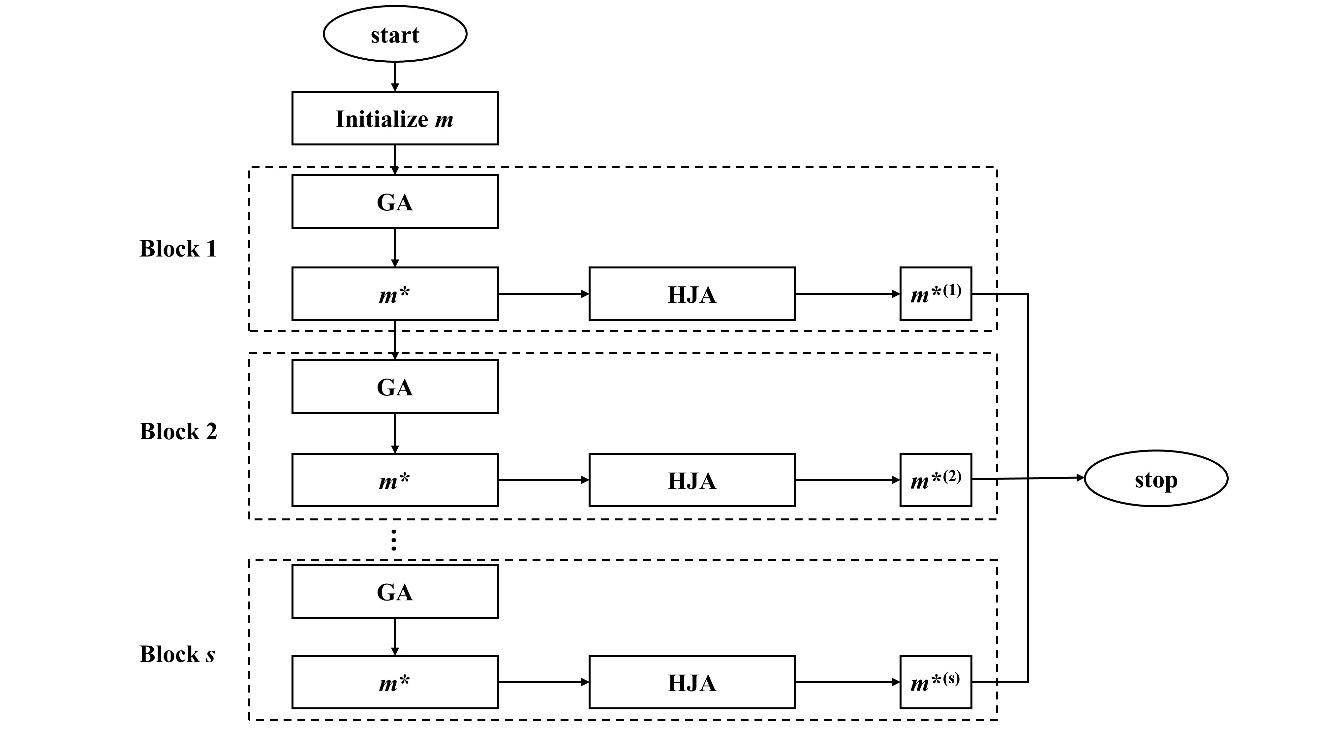
**

Fig. S3. The flow chart of combination of GA and HJA.

### S2-3. Comparison of samples designed by different algorithms

We compared the focus efficiency of samples designed by several different algorithms, including GA, HJA (which is very closed to the direct binary search that is applied in the inverse design^7^), orthogonal descent algorithm (ODA, which is applied in commercial software ZEMAX), combination of GA and HJA (our design method), combination of GA and ODA. The consuming time for each algorithm is also provided. Three cases with different parameters are considered. In first case, the diameter of lens is set as 128 μm. In second case, the diameter of lens is set as 256 μm. In third case, the diameter of lens is set as 512 μm. The number of samples in each case for each algorithm is set as 10000. In all cases the NA of samples is set as 0.1, the gray-level is set as 32, the maximum thickness is set as 1 μm and working spectrum is set as 400 nm-700 nm. The code was running on MATLAB on a server with Intel(R) Xeon(R) CPU E5-2637 v4 @ 3.50GHz. Figure S4a~S4c show the numeral distribution of samples with respect to their focus efficiency. Figure S4d~S4f show the total consumed time in each case. From these results, we can find that GA+HJA can yield samples with the highest efficiency in general, and it consumes less time compared to GA. Although applying ODA alone is the fastest method, it cannot yield high performance results, especially as the size of samples become large. This is mainly due to the fact that ODA is just a local optimization algorithm.


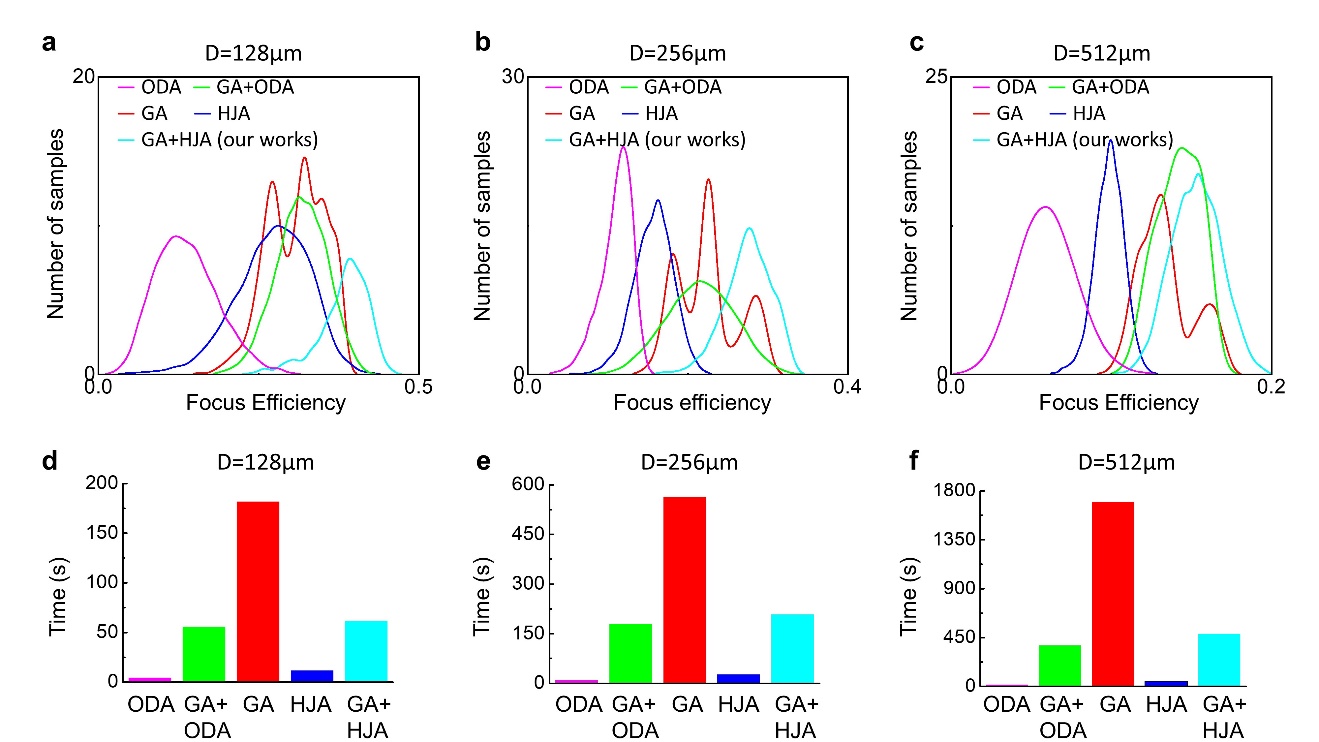


Fig. S. Comparison of samples designed by different algorithms. a~c The numeral distribution of samples with respect to focus efficiency in five cases. d~f Total consumed time in five cases.

### S2-4. Details of *Smooth* operation

In *Smooth* operation, the lens will be divided into several Fresnel zones at first. The Fresnel zone is defined as the area between two abrupt changes in the envelope of height profile, as is shown in Fig. S5a. In each zone, the structures with high aspect ratio will be eliminated. The aspect ratio *α* of each structure is defined as the ratio of the height *h*_0_ to the width Δ_0_ (i.e., *h*_0_/Δ_0_) of this structure, as is shown in Fig. S5b. The new aspect ratio α_new_ is calculated by

$\alpha_{new}=\frac{\alpha}{e^{\beta(\alpha-\alpha_{0})}+1}$ ()

where *α*_0_ is the reference aspect ratio and *β* is a parameter which is usually set as 5~10. Fig. S5c shows the ratio *α*_new_/*α* with respect to *α* under different *β* (where α_0_ is set as 2), which indicates that the aspect ratio of a structure will decrease after *Smooth* if its original aspect ratio *α*>*α*_0_. The new height *h*_new_ of each structure after *Smooth* is calculated as *h*_new_ = *α*_new_Δ_0_. Fig. S5d shows statistic results of the aspect ratio of all structures in a sample before (top) and after (bottom) *Smooth* operation, respectively. It’s clear that the number of structures with high aspect ratio (>2:1) decreases a lot. Then we test the effect of fabrication errors by apply a height variation δ*h*(*ρ*) (which assumes to be proportion to the *h*(*ρ*), corresponding to the experimental result shown in main text) to the design results, and calculate the focus efficiency, as is shown in Fig. S5e. The efficiency drops down quickly as the max δ*h* increases to 400 nm for the samples without *Smooth*. In contrast, the efficiency drops down slowly for the samples after *Smooth*.


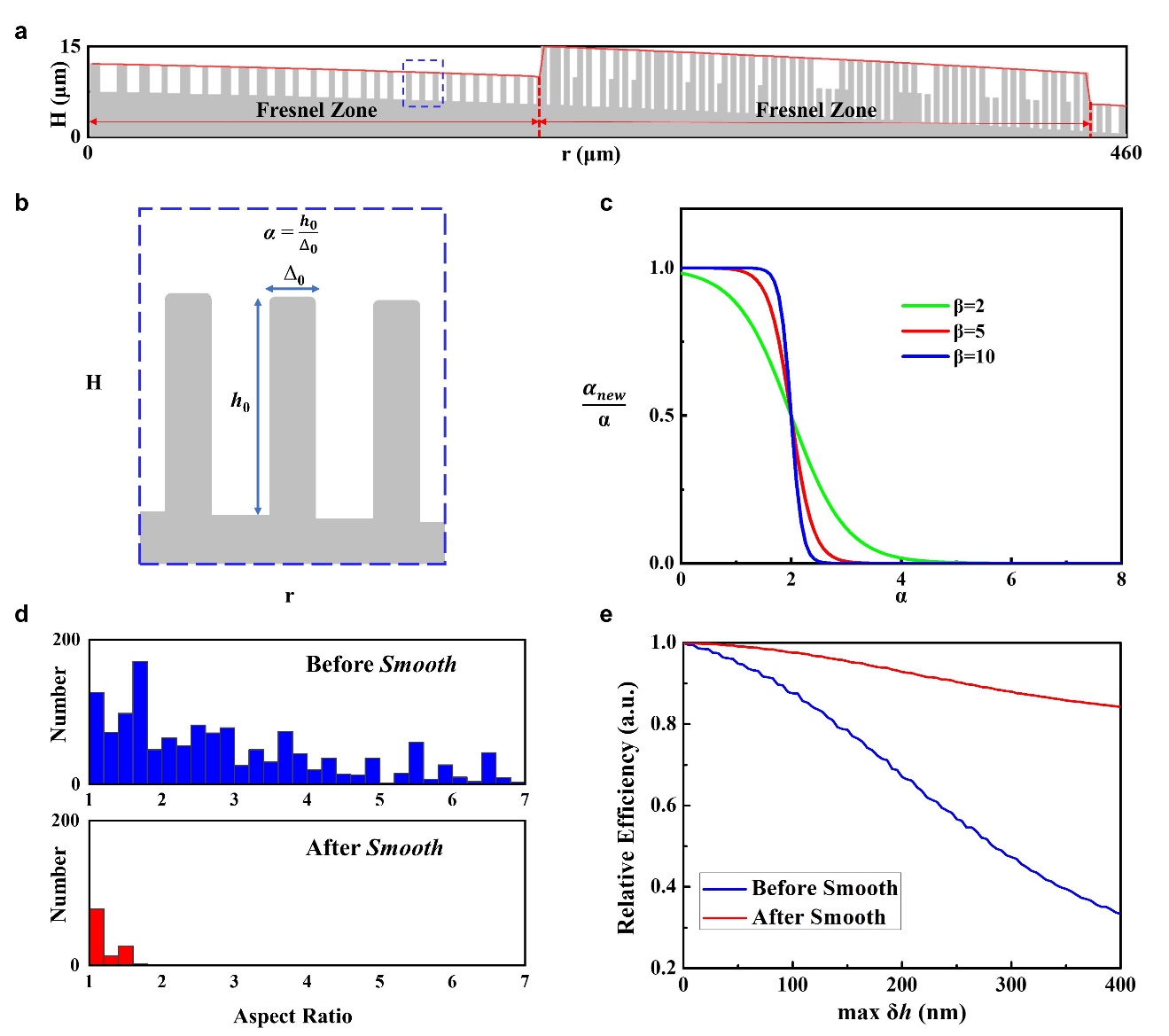


Fig. S5. Analyzation of *Smooth* operation. a The division of the zone. The red line is the envelope of the height profile. b Zoom-in images denoted by closed blue dashed lines in a. The aspect ratio *α* of the middle structure is defined as height(*h*_0_)/width(*w*_0_). c Ratio *α*_new_/*α* with respect to *α* under different *β*. d Statistical distribution diagram of the number of structures with different aspect ratios before *Smooth* (top) and after *Smooth* (bottom). e Distribution of efficiency with respect to the max height error δ*h* for samples before *Smooth* (blue line) and after *Smooth* (red line).

### S2-5. Calculated light intensity profiles along z axis for other samples


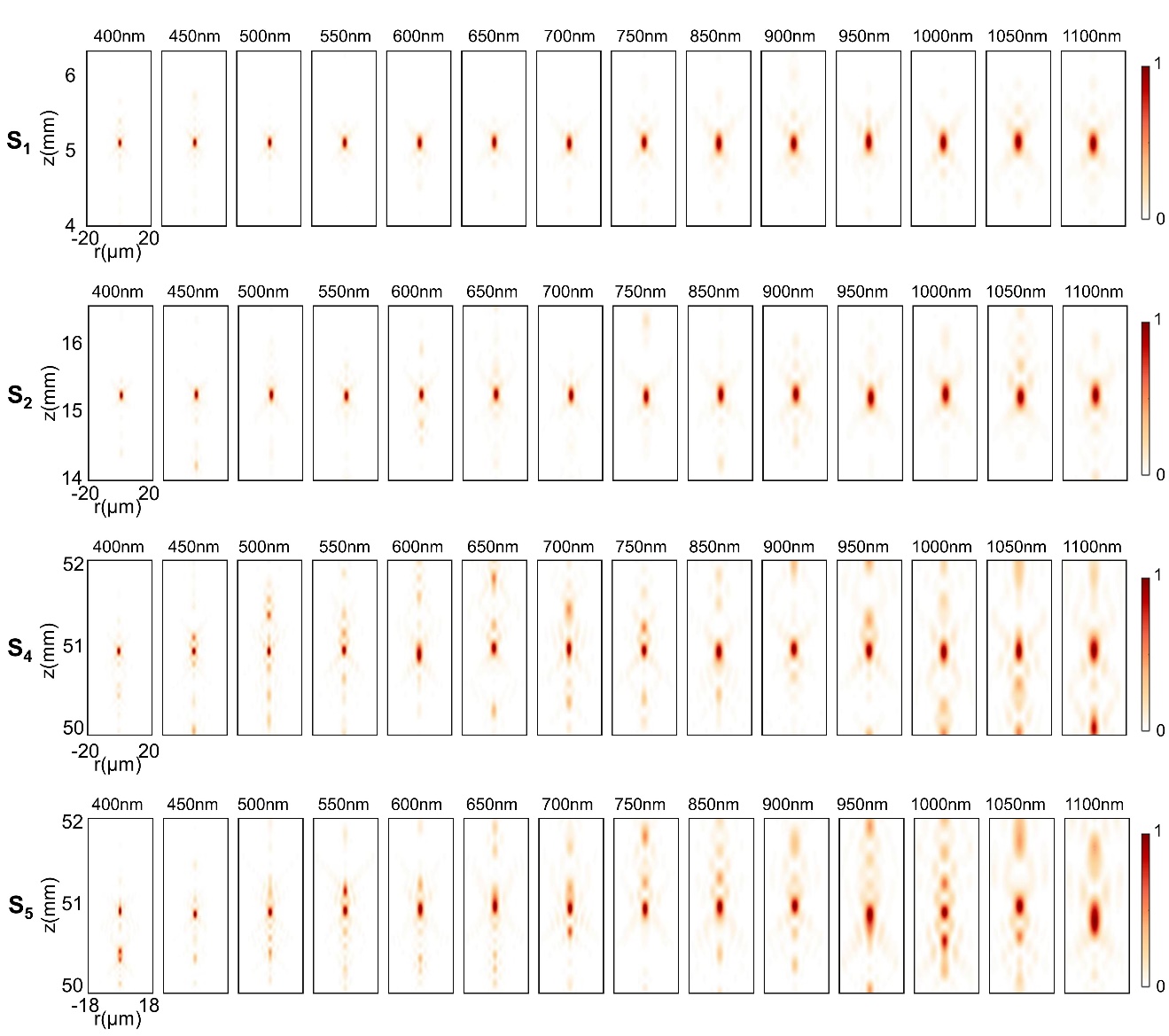


Fig. S6. Calculated light intensity profiles along propagation axis for S_1_, S_2_, S_3_, S_4_ at 14 wavelengths.

### S2-6. The effect of working bandwidth and design of a centimeter-scale AMDL with NA = 0.3

The restriction relation between working bandwidth Δ*λ* and other parameters can be derived from Eq. S15 based on numerical calculation. However, it is hard to show such high-dimensional relations (including Δ*λ*, *H*, *D*, *NA*, *J*_ω_(*F*)) entirely in a single figure. For simplicity, we fix the diameter (*D*=1cm) and *NA* (0.3), and calculate the distribution of max *J*_ω_(*F*) with respect to different Δ*λ* (ranging from 20 nm to 300 nm with the center wavelength fixed at 565 nm) and *H* (ranging from 1 μm to 35 μm), which is shown in Fig. S7a. It is clear that max *J*_ω_(*F*) will increase as Δ*λ* decreases. Fig. S7b shows the distribution of max *J*_ω_(*F*) with respect to *H* where the working spectrum is fixed at 450-680 nm (denoted by orange dashed lines in Fig. S7a). The experimental results in the main text reveal that to design an AMDL with relatively good performance, the max *J*_ω_(*F*) of such an AMDL need to be at least 0.2. Therefore, the thickness *H* is chosen as 28 μm as is shown in Fig. S7b. The optimal height distribution of an AMDL with D = 10010 μm, NA = 0.3, *H* = 28 μm and working spectrum from 450 nm to 680 nm is designed by the optimization framework, as is shown in Fig. S7c. In the design, the width of ring is set as 0.7 μm to satisfy Nyquist−Shannon sampling theorem, which means the max aspect ratio will reach 40:1. Thanks to the *Smooth* operation, there exist little high aspect ratio structures in the final distribution, as is depicted in Fig. S7d. Figure S7e shows the calculated light intensity profiles along propagation axis for this sample at 24 wavelengths, which confirm the achromatic properties.


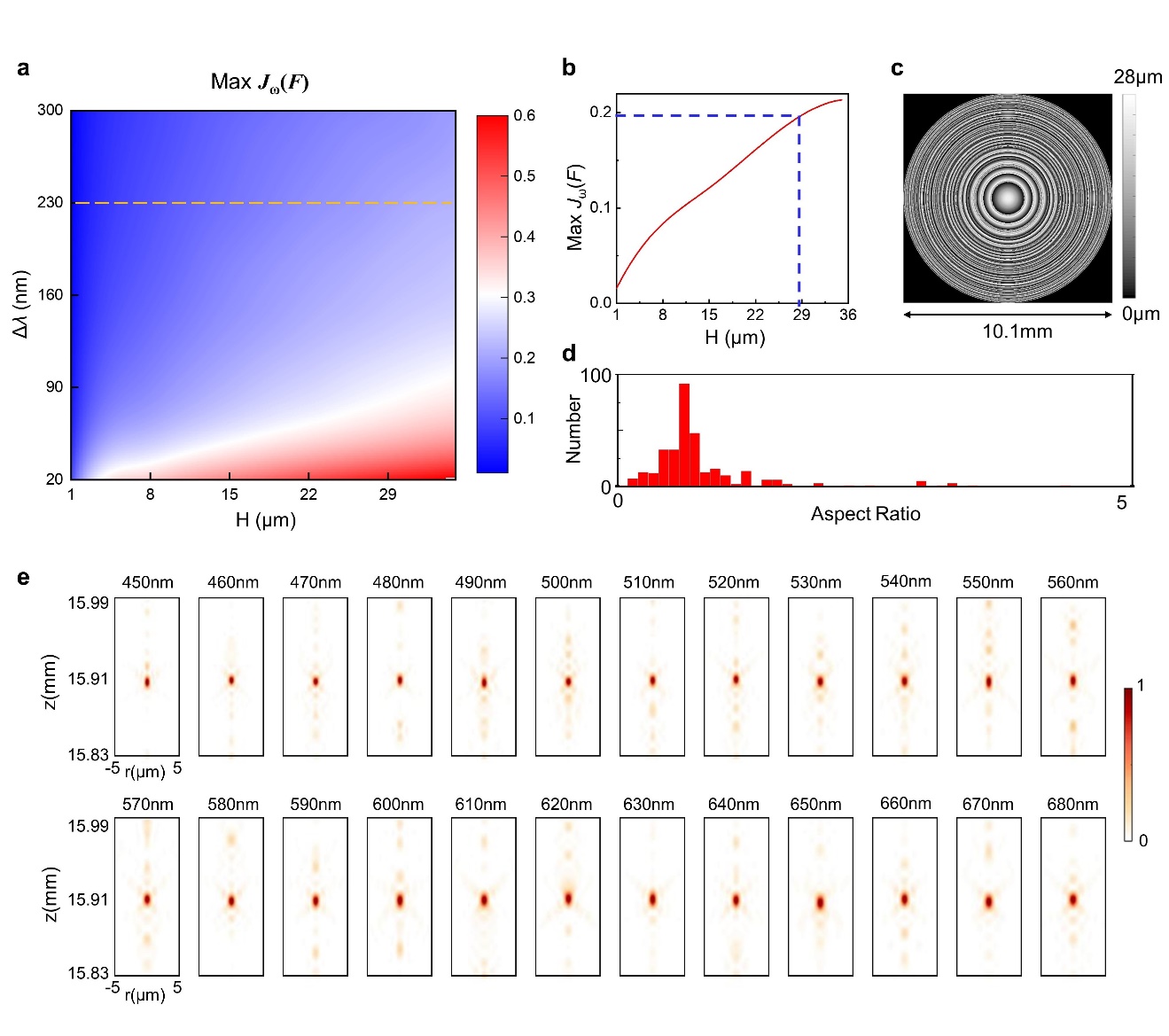


Fig. S. The effect of working bandwidth and design of a centimeter-scale AMDL with NA = 0.3. a The distribution of max *J*_ω_(*F*) with respect to different samples in dimensions of working bandwidth and thickness. The orange dashed lines denote the distribution where the working spectrum is fixed at 450-680 nm. b Max *J*_ω_(*F*) of the AMDL with *H* ranging from 1μm to 35 μm and the working spectrum fixed at 450-680 nm. c Height distribution (2D top view) of this AMDL. d Statistical distribution diagram of the number of structures with different aspect ratios in the AMDL. e Calculated light intensity profiles along propagation axis for the AMDL at 24 wavelengths.

### Additional experimental results

### S3-1. Refractive index of AZ4562


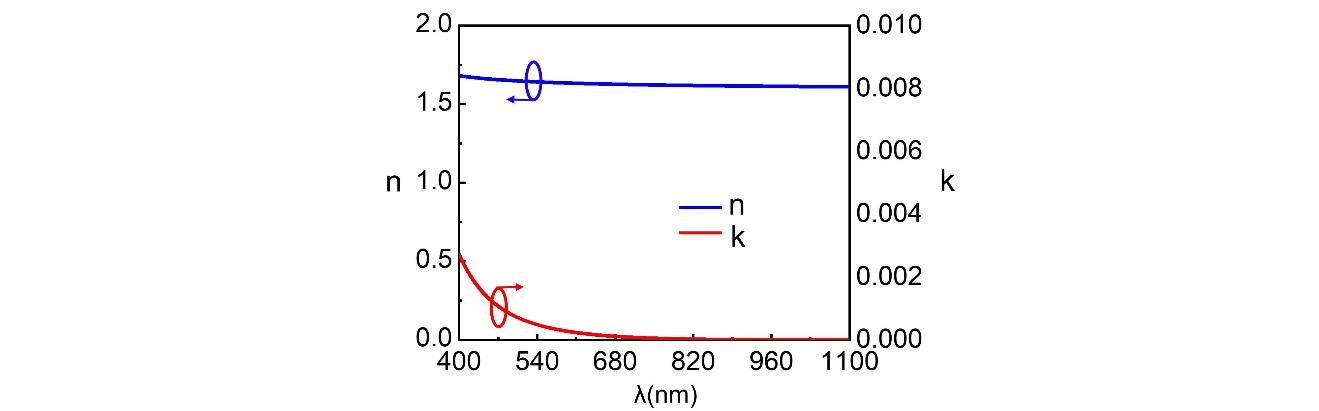


Fig. S. Real part (n) and imaginary part (k) of refractive index of AZ4562.

### S3-2. Photographs of other samples


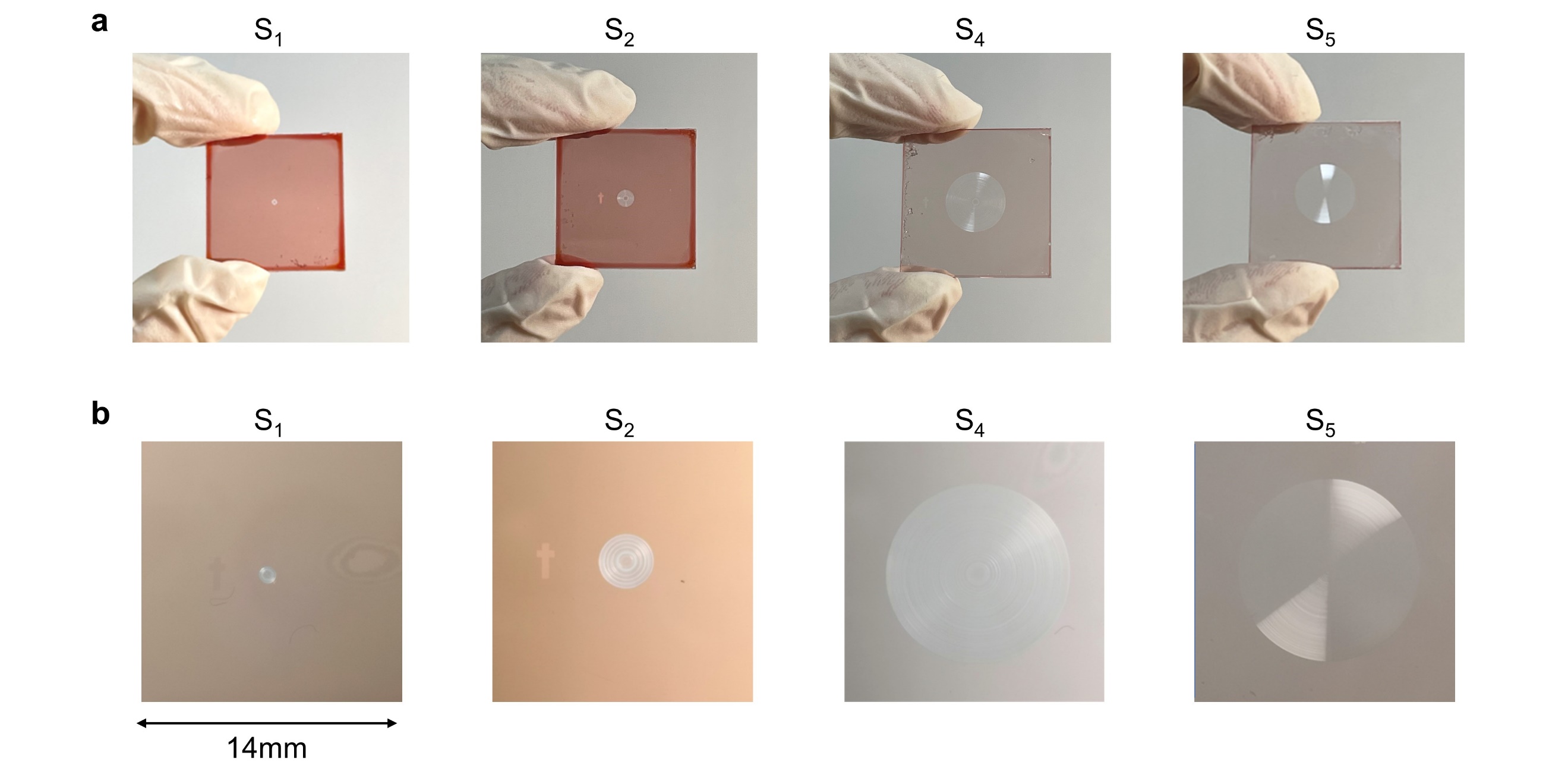


Fig. S. Photographs of other samples. a Photographs of fabricated samples S_1_, S_2_, S_4_, S_5_. b Local view of the whole AMDLs.

### S3-3. Photographs of the commercial refractive lens and Fresnel lens

Photographs of the commercial refractive lens (LE1234) is shown in Fig. S10a~S10b with diameter equal to 25.4mm, focus length equal to 100mm and max thickness equal to 3.6mm. Photographs of the commercial Fresnel lens is shown in Fig. S10c~S10d with diameter equal to 10mm, focus length equal to 50mm. The height profile of Fresnel lens is measured by a step profiler. The max thickness of Fresnel lens used to accumulate optical path is about 40 μm.

**
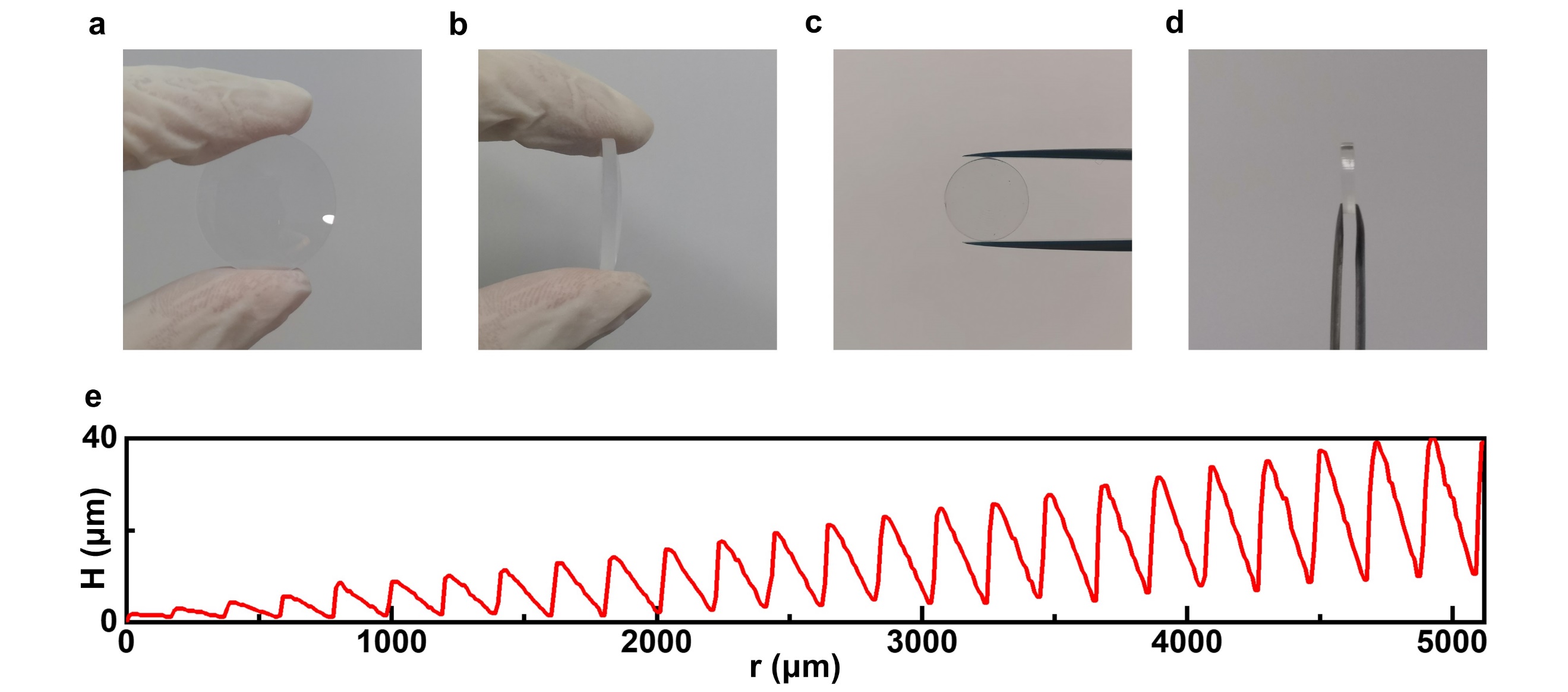
**

Fig. S. Photographs of the commercial refractive lens and Fresnel lens. a Front view of the refractive lens. b Side view of the refractive lens. c Front view of the Fresnel Lens. d Side view of the Fresnel Lens. e Height distribution of the Fresnel lens along the radial axis.

### S3-4. Optical configurations


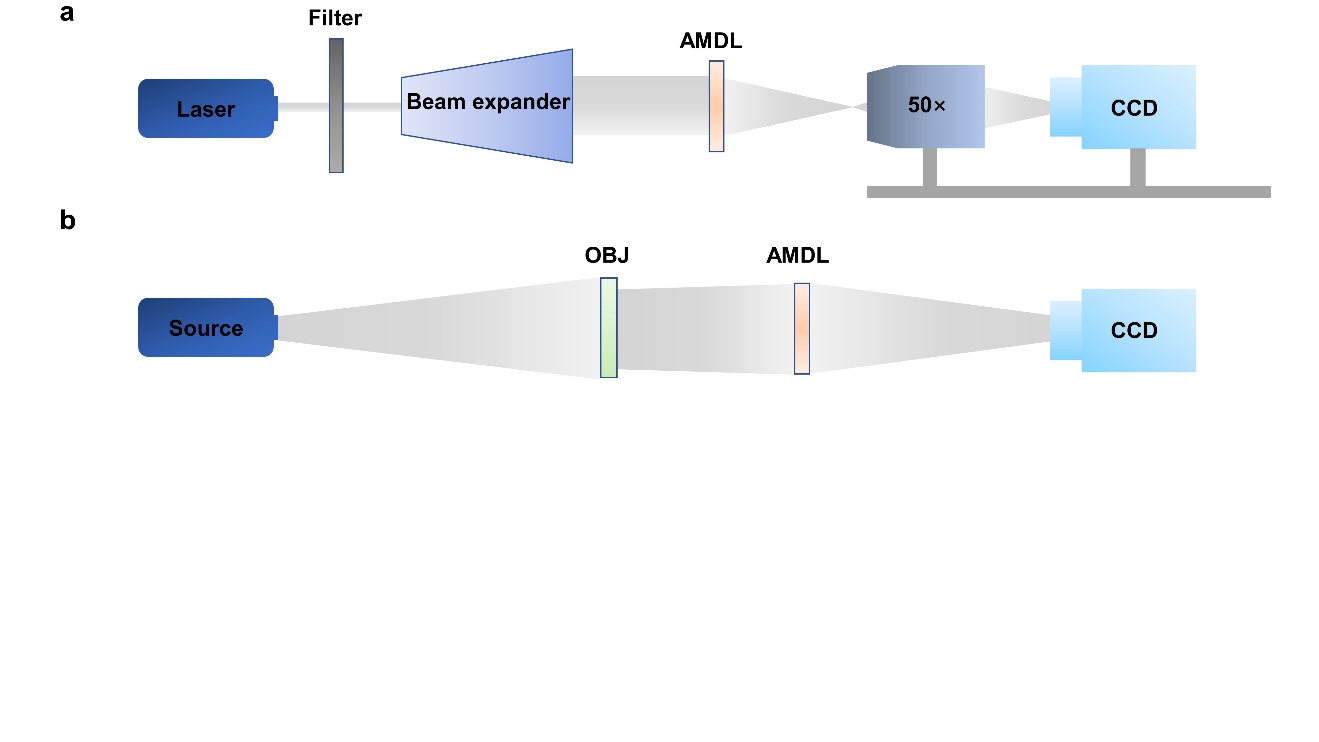


Fig. S. Optical configurations for experimentally verifying the performance of AMDL and Fresnel lens. a Configuration for measuring focus length and light intensity distributions on focus plane, where an objective (50×magnification, NA = 0.42) is used. b Configuration for imaging, where OBJ denotes different imaging targets.

### S3-5. Spectrum of all light sources used in experiments

**
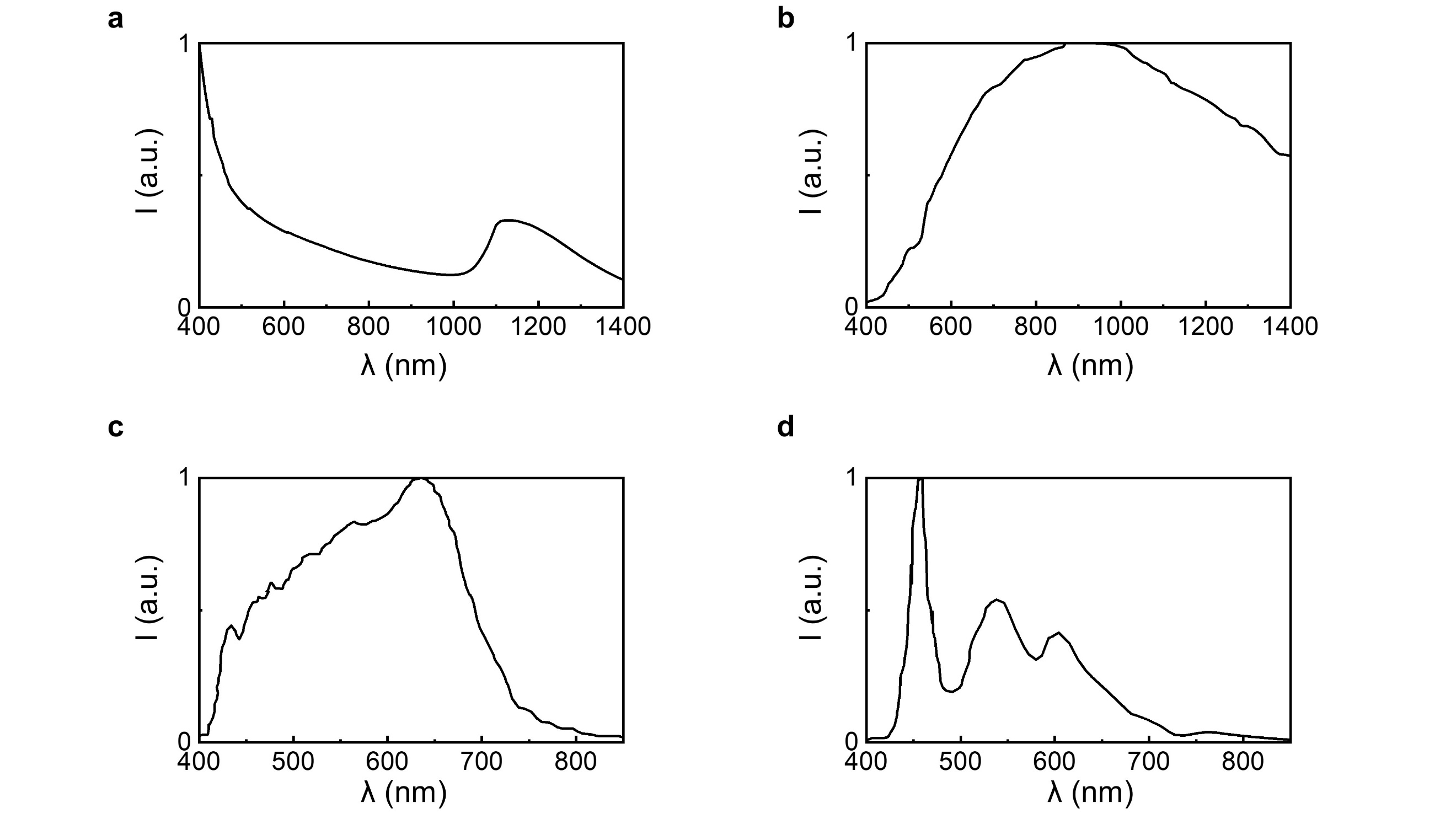
**

Fig. S. Normalized intensity of light source measured by an optical power meter. a Supercontinuous laser (Fianium). b Halogen lamp (HL100S). c Fluorescent lamp. d LED Projector. The monochromatic light is generated by combining the Fianium with different filters, of which the bandwidth is ~10 nm.

### S3-6. Focus performance of other samples

Figure S13~S16 show the measured cross-sectional intensity profiles (top) and measured 2D PSF (bottom) at 14 different wavelengths for sample S_1_, S_2_, S_4_, S_5_, respectively. The Strehl ratio calculated based on these PSFs are already shown in Fig. 4f in the main text. It should be mentioned that part of light would diffract in large angle and may not be captured by the CCD, especially for the AMDL with low focus efficiency (such as S_5_). To eliminate the effect of this phenomenon, an alternative method is to multiply origin Strehl ratio by the ratio of power in the Airy-radius to the power in the whole focus plane (measured by the optical power meter), which (called Normalized Strehl ratio) is shown in Fig. S17. The average value for S_1_~S_5_ is 0.6614, 0.4901, 0.2811, 0.1402, 0.0515, respectively.

**
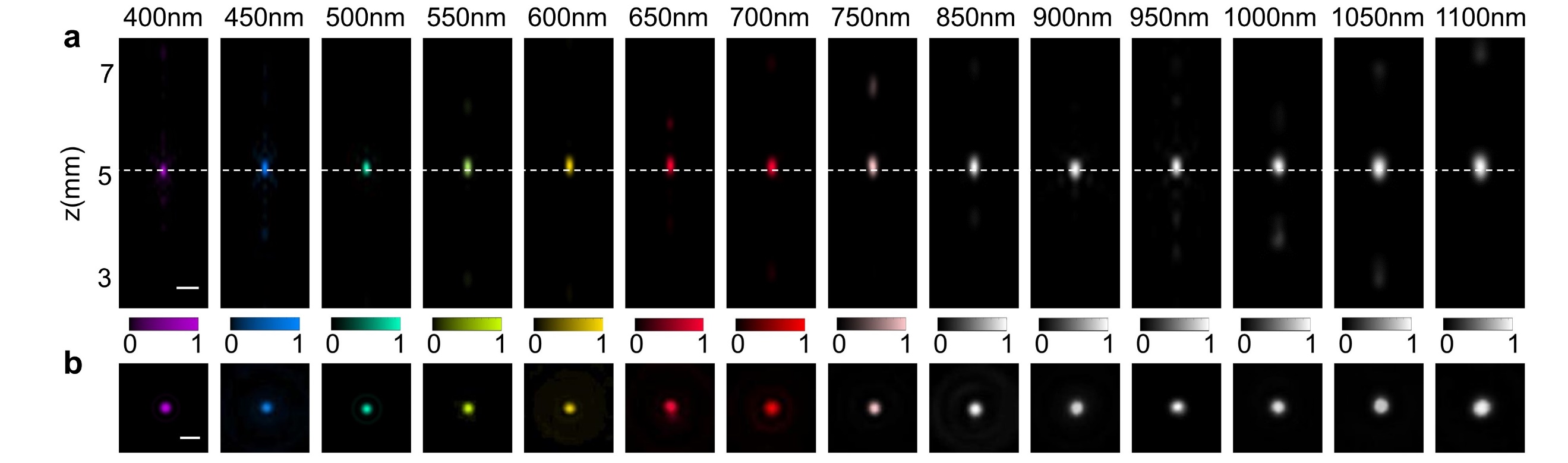
**

Fig. S. Measured achromatic focusing characteristic of S_1_. a Light intensity profiles along the propagation at 14 different wavelengths. Scale bars, 15 μm. b Normalized intensity profiles along the white dashed lines of a. Scale bars, 10 μm.

**
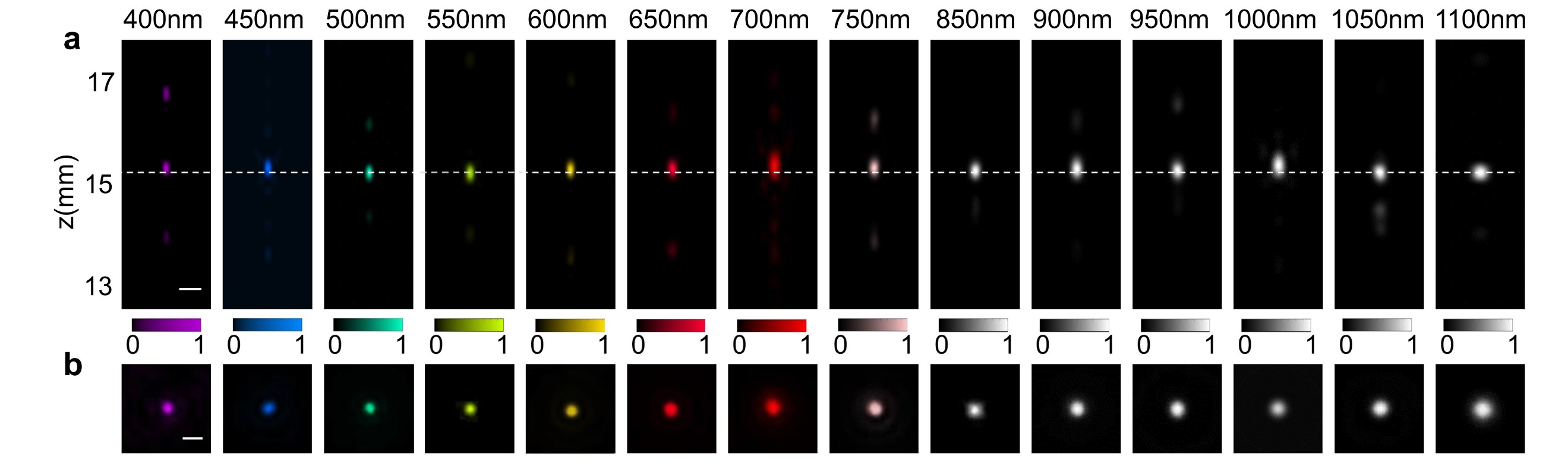
**

Fig. S. Measured achromatic focusing characteristic of S_2_. a Light intensity profiles along the propagation at 14 different wavelengths. Scale bars, 15 μm. b Normalized intensity profiles along the white dashed lines of a. Scale bars, 10 μm.

**
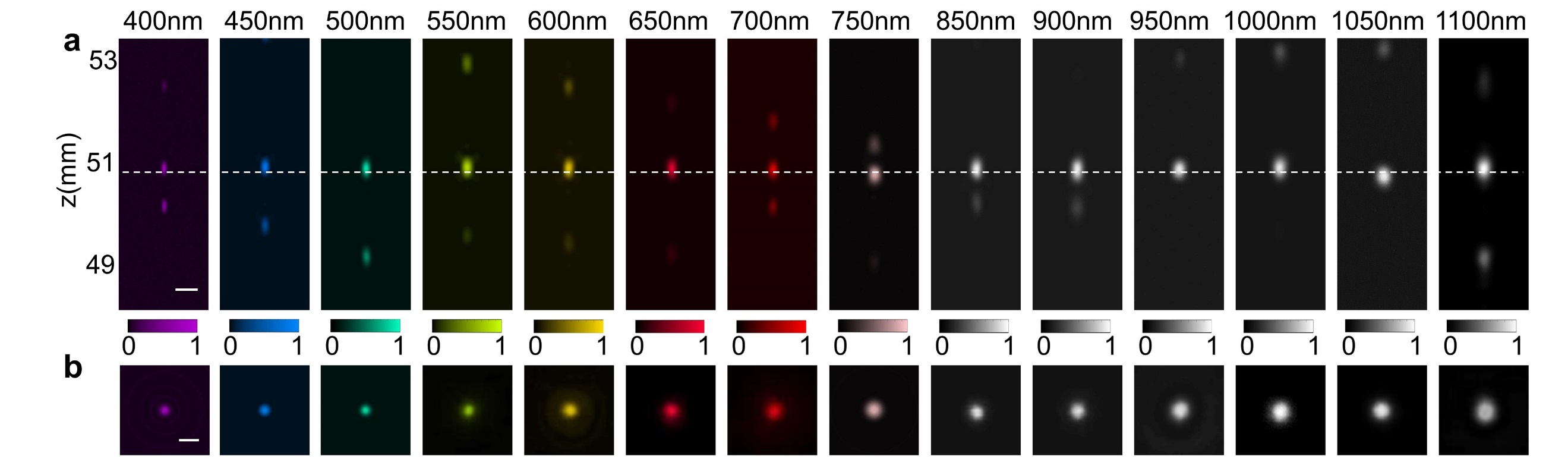
**

Fig. S. Measured achromatic focusing characteristic of S_4_. a Light intensity profiles along the propagation at 14 different wavelengths. Scale bars, 15 μm. b Normalized intensity profiles along the white dashed lines of a. Scale bars, 10 μm.

**
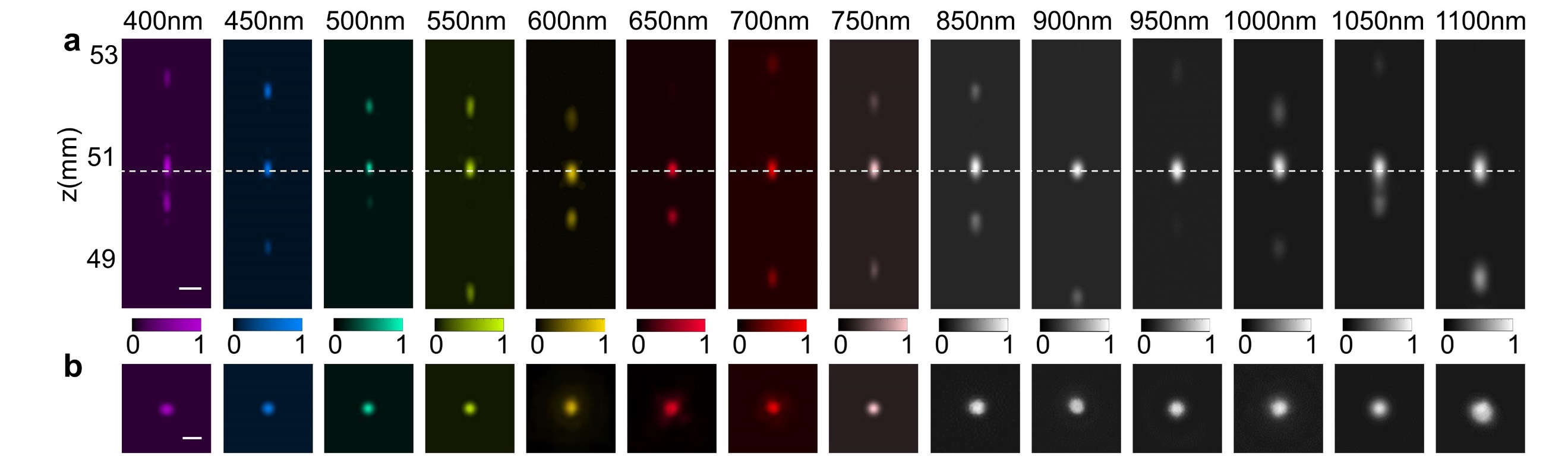
**

Fig. S. Measured achromatic focusing characteristic of S_5_. a Light intensity profiles along the propagation at 14 different wavelengths. Scale bars, 15 μm. b Normalized intensity profiles along the white dashed lines of a. Scale bars, 10 μm.


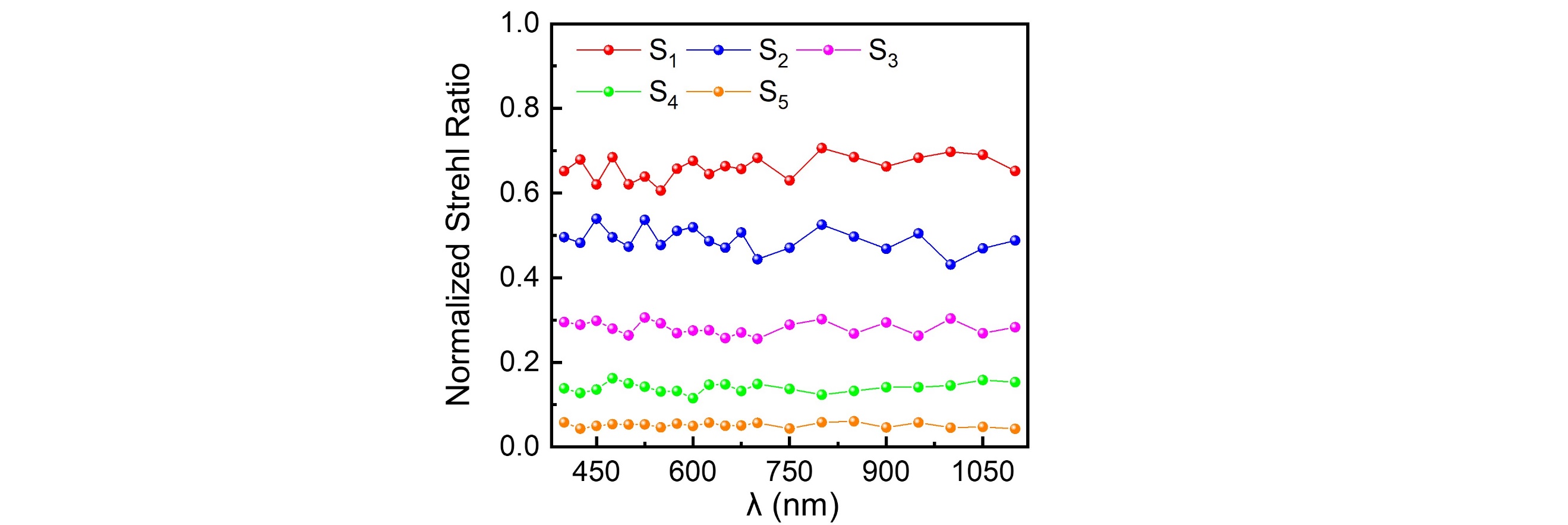


Fig. S17. Normalized Strehl ratio of S_1_~S_5_. Strehl Ratio of S_1_~S_5_ after considering the ratio of power in Airy-radius to the power in the whole focus plane.

### S3-7. Comparison of images taken from Fresnel lens, refractive lens and AMDL

Figure S18 shows the comparison between images taken from Fresnel lens, refractive lens and AMDL S_3_ under white light illuminance. Color blurred effect do exist in the image taken from the Fresnel lens and the refractive lens due to the chromatic aberration while it does not exist in the image taken from AMDL S_3_ due to achromatic design.

**
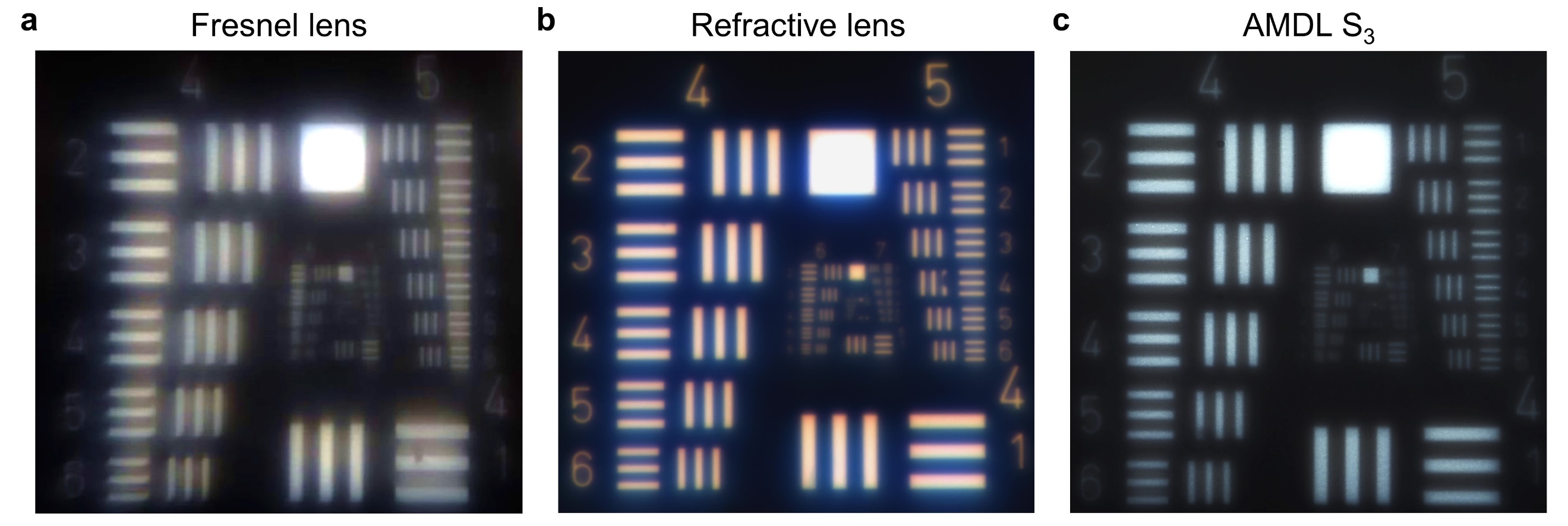
**

Fig. S. Comparison between images taken from Fresnel lens, refractive lens and AMDL S_3_ under broadband white light illumination. a Image of 1951 USAF resolution test chart taken from the Fresnel lens. b Image of 1951 USAF resolution test chart taken from the refractive lens. c Image of 1951 USAF resolution test chart taken from AMDL S_3_.

### S3-8. Calculation of Broadband MTF

We apply two methods to calculate the MTF of AMDL. One is based on PSF on the focus plane, and the other is based on Edge Spread Function (ESF) at the same place. The broadband MTF is calculated based on measured PSF and ESF of AMDL under white light illuminance (400~800 nm), as is shown in Fig. S19a and Fig. S19b for sample S_3_, respectively. Fig. S19c shows the MTFs calculated by PSF (red line) and ESF (blue line), respectively. The MTF calculated by ESF oscillates as spatial frequency increases, which is mainly due to the fact that the light source is not perfectly uniform and causes the ESF not uniform along the edge. Nevertheless, two results are closed to each other, which means the measured MTF is comparatively accurate. The broadband MTF of ideal achromatic lens is calculated based on ideal broadband PSF, which is calculated by integrating the diffraction limited PSF over the whole spectrum.


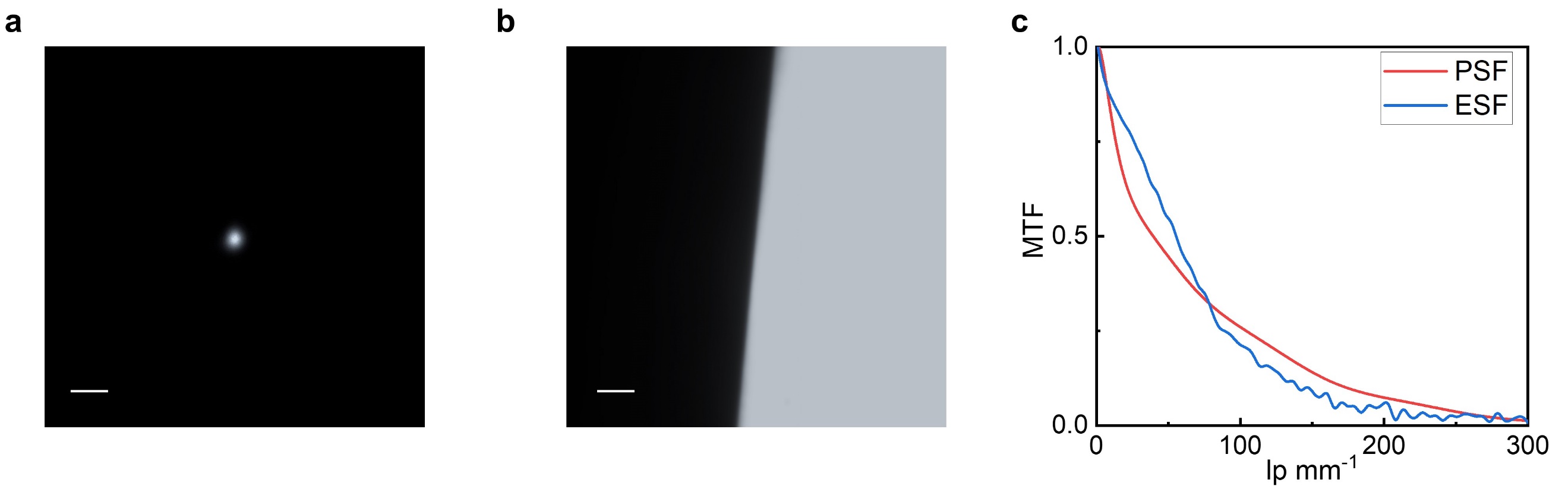


Fig. S. Calculation of Broadband MTF. a PSF for sample S_3_ under broadband incoherent illumination. Scale bar, 15 μm. b ESF for sample S_3_ under broadband incoherent illumination. Scale bar, 50 μm. c MTF calculated by two different methods. Red line and blue line are MTF calculated by PSF and ESF, respectively.

### S3-9. Imaging processing by using built-in algorithm of cell phones

By using the built-in image processing in a cell phone (HUAWEI, VCE-AL00), we can reconstruct high-contrast images from raw images taken by AMDLs with steps below. Select a photo in cell phone, click Edit – Adjust – Contrast or Sharpness or Shadows, and change the level of corresponding characters of the photo. We set contrast at level 5, sharpness at level 10 and shadows at level -10 in the following as an example. Figure S20a show the images of USAF taken from S_1_~S_5_ before and after processing. Figure S20b depicts the image contrast of raw images and images after processing. The image contrast of reconstructed images will almost reach the maximum +1 for S_1_~S_3_, while it is even below 0.2 for S_5_. Figure S20c show the image of checkerboard taken by AMDL S_3_ and Fresnel lens, where a Fluorescent lamp is used as the illumination source. The results after imaging processing indicate that the color blurred effect cannot be removed in the process. These results highlight the necessity of achromatic design and reservation of details in raw images, and further demonstrate that the comprehensive performance of sample S_3_ is the best on in five samples.


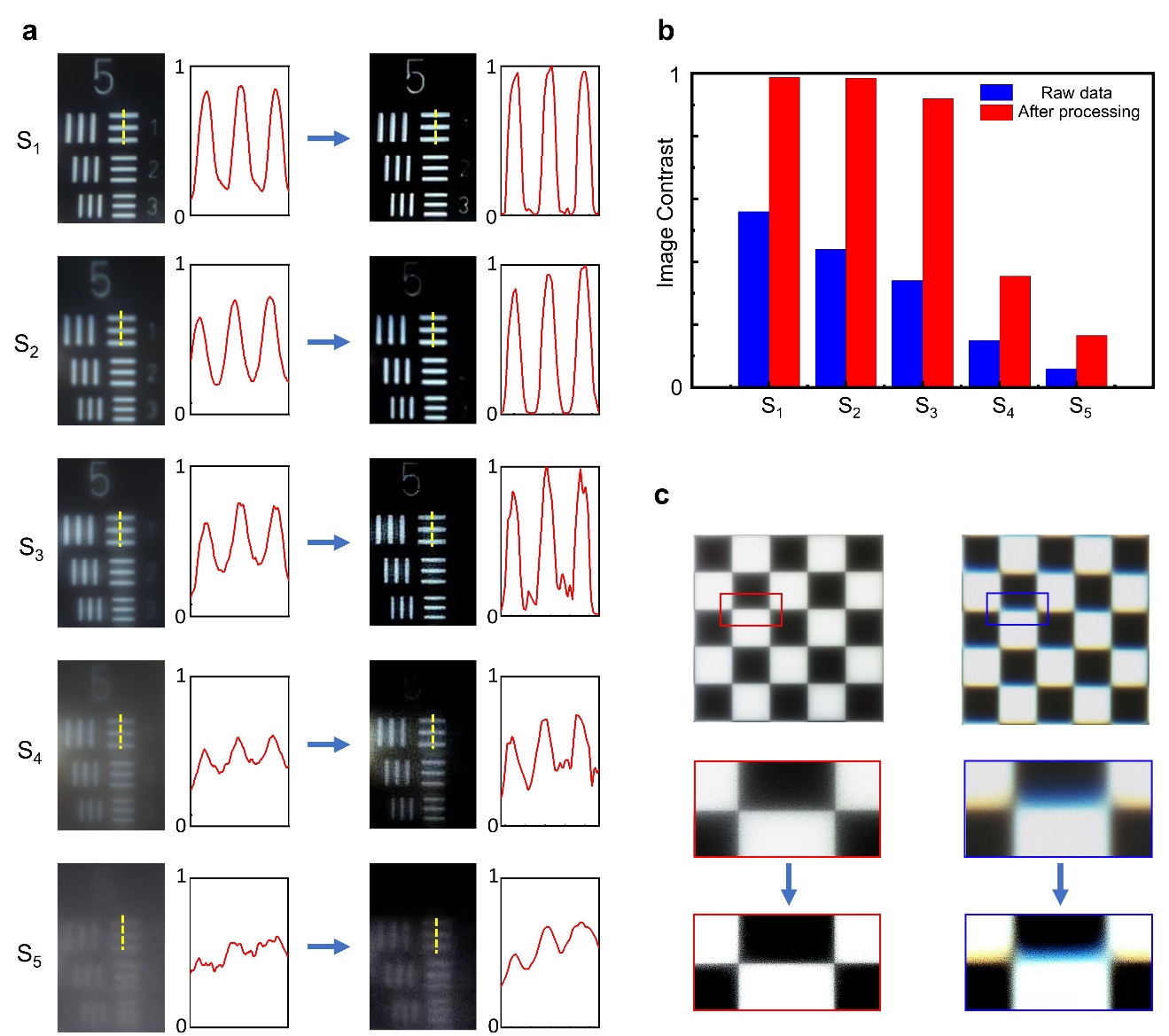


Fig. S. Imaging processing. a Image results of 1951 USAF resolution test chart taken from S_1_ ~ S_5_ before processing (left) and after processing (right). b Image contrast before and after imaging processing. c Image results of checkerboard taken by AMDL S_3_(left) and Fresnel lens(right). The bottom one is the local view and corresponding image after processing.

**Reference**

1. Born, M. & Wolf, E. *Principles of Optics*: *Electromagnetic Theory of Propagation, Interference and Diffraction of Light*. (Elsevier Science, 2013), pp. 554-632.
2. Goodman, J. W. *Introduction to Fourier Optics.* (McGraw-Hill, San Francisco, 1968), pp. 38-95.
3. Shrestha, S., Overvig, A. C., Lu, M., Stein, A. & Yu, N. Broadband achromatic dielectric metalenses. *Light Sci. Appl.* 7, 85 (2018).
4. Haupt, R. L. & Werner, D. H. *Genetic Algorithms in Electromagnetics.* (Wiley, IEEE-press, Hoboken, 2007)
5. Hooke, R. & Jeeves, T.A. Direct search solution of numerical and statistical problems. *Journal of the ACM* 8, 212–229 (1961).
6. Nocedal, J. & Wright, S. J. *Numerical Optimization.* (Springer, New York, 2006).
7. Meem, M. et al. Imaging from the visible to the longwave infrared wavelengths via an inverse-designed flat lens. *Opt. Express* 29, 20715-20723 (2021).
